# Sortase-mediated segmental labeling: A method for segmental assignment of intrinsically disordered regions in proteins

**DOI:** 10.1371/journal.pone.0258531

**Published:** 2021-10-28

**Authors:** Kristina V. Boyko, Erin A. Rosenkranz, Derrick M. Smith, Heather L. Miears, Melissa Oueld es cheikh, Micah Z. Lund, Jeffery C. Young, Patrick N. Reardon, Mark Okon, Serge L. Smirnov, John M. Antos

**Affiliations:** 1 Department of Chemistry, Western Washington University, Bellingham, Washington, United States of America; 2 Department of Biology, Western Washington University, Bellingham, Washington, United States of America; 3 Oregon State University NMR Facility, Oregon State University, Corvallis, Oregon, United States of America; 4 Department of Biochemistry and Molecular Biology, Department of Chemistry, Michael Smith Laboratories, University of British Columbia, Vancouver, British Columbia, Canada; Rijksuniversiteit Groningen, NETHERLANDS

## Abstract

A significant number of proteins possess sizable intrinsically disordered regions (IDRs). Due to the dynamic nature of IDRs, NMR spectroscopy is often the tool of choice for characterizing these segments. However, the application of NMR to IDRs is often hindered by their instability, spectral overlap and resonance assignment difficulties. Notably, these challenges increase considerably with the size of the IDR. In response to these issues, here we report the use of sortase-mediated ligation (SML) for segmental isotopic labeling of IDR-containing samples. Specifically, we have developed a ligation strategy involving a key segment of the large IDR and adjacent folded headpiece domain comprising the C-terminus of *A*. *thaliana* villin 4 (AtVLN4). This procedure significantly reduces the complexity of NMR spectra and enables group identification of signals arising from the labeled IDR fragment, a process we refer to as *segmental assignment*. The validity of our segmental assignment approach is corroborated by backbone residue-specific assignment of the IDR using a minimal set of standard heteronuclear NMR methods. Using segmental assignment, we further demonstrate that the IDR region adjacent to the headpiece exhibits nonuniform spectral alterations in response to temperature. Subsequent residue-specific characterization revealed two segments within the IDR that responded to temperature in markedly different ways. Overall, this study represents an important step toward the selective labeling and probing of target segments within much larger IDR contexts. Additionally, the approach described offers significant savings in NMR recording time, a valuable advantage for the study of unstable IDRs, their binding interfaces, and functional mechanisms.

## Introduction

Intrinsically disordered regions (IDRs), often involving hundreds of residues, are widespread in all kingdoms of life and perform many key cellular roles [1–5]. These disordered polypeptides are dynamic systems, wherein there exists a set of distinct semi-structured or semi-disordered states for each IDR. Moreover, the balance between these states can be altered by changes in the environment, posttranslational modifications, and binding to other domains or molecules, which in turn impacts IDR function [6, 7]. This inherent complexity makes IDR systems difficult to probe experimentally. This challenge is heightened by other factors, such as the proteolytic instability of IDRs in solution, as well as the fact that IDRs with posttranslational modifications are often present as chemically heterogeneous mixtures. Thus, there is a growing need for new experimental approaches for characterizing IDRs, including their functions and binding interfaces.

Methods for the investigation of IDRs commonly include NMR spectroscopy, FRET and others, provided that the appropriate probes can be inserted into the IDR sequence (e.g. fluorophores or NMR-active isotopic labels) [8–10]. Of these, modern NMR is a uniquely powerful tool for site-specific studies of conformational ensembles, dynamics, function and binding mechanisms of IDRs [11–17]. However, in addition to the IDR instability noted above, using NMR to study larger IDRs (>100 residues) can be prohibitively challenging due to severe spectral overlap and resonance assignment issues [18]. Several sophisticated residue-specific NMR resonance assignment approaches have been described for larger IDRs (>100 residues) [19–21]. While offering exceptional resolving power for large IDRs, the implementation of these and related techniques require a combination of high magnetic field NMR spectrometers equipped with expensive cryoprobes, and for specially trained and experienced personnel to setup and execute such pulse programs appropriately. Moreover, these approaches rely on relatively time-consuming 3D/4D/5D NMR approaches for the NMR resonance assignment of large IDRs, often requiring several days of recording time. This, in combination with other factors, may make such methods less practical for many IDRs due to their limited stability and lifetime (often under 24 hours).

Given the difficulties listed above, a complementary alternative to more sophisticated and time-consuming 3D/4D/5D schemes is desirable and could allow a much broader user base to probe sizable IDRs by NMR. To this end, segmental labeling with NMR-active nuclei has the potential to circumvent many of these challenges by reducing spectral overlap and enabling inherently faster 2D NMR schemes. This could allow for meaningful, segment-specific identification of IDR resonances and their usage as functionally relevant probes. It should be noted that segmental labeling options for protein NMR also offer the advantage of allowing production of large cytotoxic samples from smaller non-toxic components, which is a feature that has been reviewed elsewhere and may be particularly relevant for large IDRs [22]. Despite these apparent advantages, the application of segmental labeling to proteins with sizable IDRs remains underdeveloped and is only addressed by a handful of studies (including some recent ones), with IDRs not always being the target of interest [23–27].

A growing number of strategies are available for protein segmental labeling, the majority of which rely on native chemical ligation (NCL) / expressed protein ligation (EPL) or protein trans splicing (PTS) methodology [28–33]. In addition, approaches utilizing enzymes such as the well-studied sortases [26, 27, 34–38] and the more recently reported asparaginyl endopeptidases have emerged [39, 40]. These enzymatic methods are largely unexplored in the context of characterizing IDRs via NMR, and to our knowledge only two examples of applying sortase to the segmental isotopic labeling of proteins containing significant IDRs have been reported [26, 27]. Despite its limited usage in the context of IDRs, a sortase-based approach offers compelling advantages such as the fact that it operates *in vitro* under mild reaction conditions, requires modest alterations to the polypeptide target, and relies on easily accessible sortase enzymes [41, 42]. The continued development of this technique could therefore provide a much-needed complement to NCL/EPL/PTS, enabling new spectroscopic studies of IDRs and offering alternatives where NCL/EPL/PTS may not be optimal.

**Fig 1:**
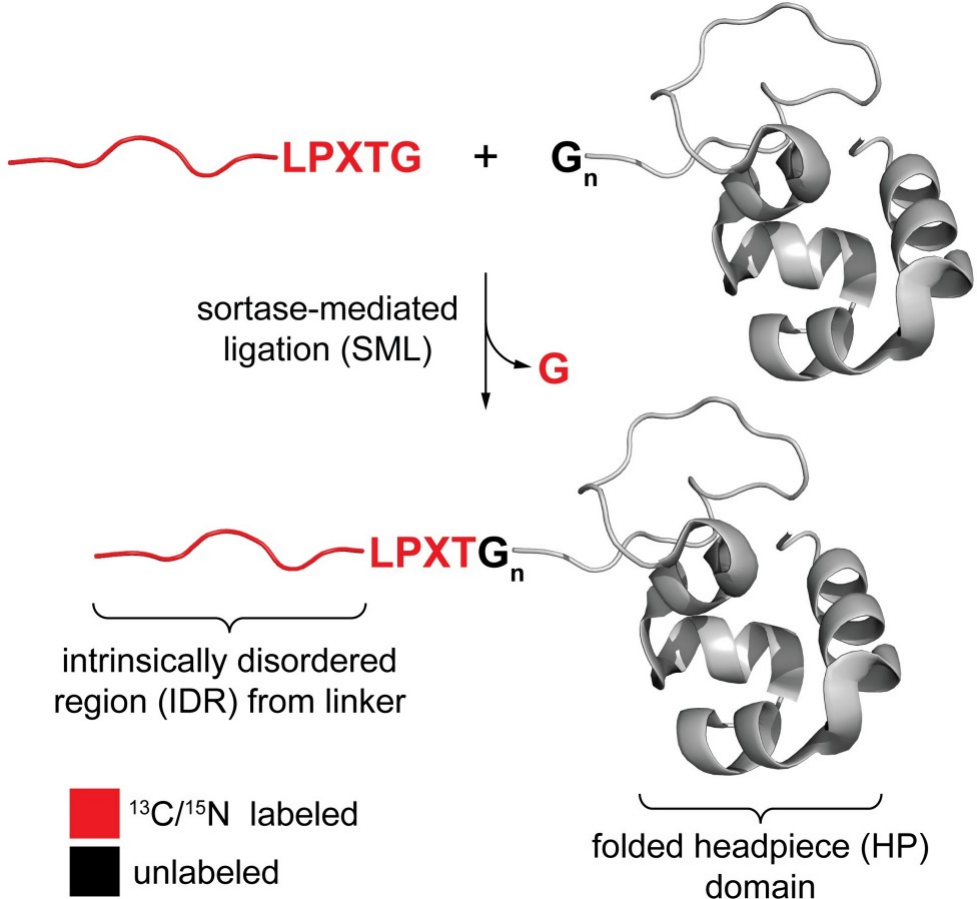
Segmental isotopic labeling via Sortase-Mediated Ligation (SML). Strategy for segmental isotopic labeling of villin 4 utilizing SML between the disordered linker and folded headpiece. LPXTG represents the requisite sortase recognition site.

Here we employ sortase-mediated ligation (SML) for segmental isotopic labeling and assignment of a key IDR fragment from the plant cytoskeleton regulator villin 4 (**Fig 1**). This protein is a member of a superfamily encompassing supervillin, dematin and plant villins, where each protein contains a large IDR (~130–830 residues) of significant functional value [18, 43, 44]. Although supervillin, dematin and plant villins are very relevant study targets due their central cellular roles, they remain relatively undercharacterized in comparison to related proteins without large IDRs (e.g., vertebrate villin and gelsolin). In the present work, we have used SML to incorporate NMR-active isotopes (^15^N alone or in combination with ^13^C) into all of the residues of the acidic, 35-residue C-terminal portion of the *A*. *thaliana* villin 4 IDR while keeping the headpiece portions of the protein unlabeled. This allowed us to perform a segmental assignment of the IDR fragment of interest, in which the NMR resonances of the IDR were treated as a collective of peaks originating from the segment of interest in the sample. The validity of this group assignment was assessed through residue-specific assignment of the same IDR segment within a larger labeled context. This validation confirmed that segmental assignment provides an expedient means of probing IDRs within larger protein systems. Finally, using segmental labeling and assignment, we were able to demonstrate that the villin 4 IDR fragment is sensitive to alterations in temperature, which is a central environmental parameter for plant biochemistry and physiology. Overall, this work demonstrates the potential of SML for characterizing sizable IDRs, augmenting the existing repertoire of NMR methods for these systems. This work also provides new insight into the properties of a key IDR fragment from plant villins.

## Materials and methods

### Chemical and reagents

Triglycine peptide (GGG) was purchased from Chem-Impex (cat# 04555) and diglycine peptide (GG) was obtained from Sigma (#G1002). Ammonium chloride (15N, 99%, cat# NLM-467) and D-glucose (U-13C6, 99%, cat# CLM-1396) for isotopic labeling were purchased from Cambridge Isotope Laboratories. Deuterium oxide (D, 99.9%) for NMR recordings was also obtained from Cambridge Isotope Laboratories (cat# DLM-4). Water used in biological procedures or as a reaction solvent was purified using a Milli-Q Advantage A10 system (Millipore). All other chemicals and reagents were obtained from commercial sources and used without further purification.

### FPLC

Protein separations by size exclusion chromatography (SEC) were performed on an ÄKTAprime plus FPLC system (GE Healthcare) equipped with a HiPrep^TM^ 16/60 Sephacryl^TM^ S-100 HR column using 20 mM PIPES (pH 6.8), 50 mM NaCl buffer as the eluent at 0.5–0.7 mL/min.

### Mass spectrometry

Liquid chromatography electrospray ionization mass spectrometry (LC-ESI-MS) analyses were carried out using an Advion CMS expression^L^ mass spectrometer and a Dionex Ultimate 3000 HPLC system equipped with a Phenomenex Aeris 3.6 μm WIDEPORE C4 200 Å column (100 x 2.1 mm). Separations upstream of the mass spectrometer were achieved with an aqueous (95% H_2_O, 5% MeCN, 0.1% formic acid) / organic (MeCN, 0.1% formic acid) mobile phase at 0.3 mL/min (method: hold 10% organic 0.0–1.0 min, linear gradient of 10–90% organic 1.0–7.0 min, hold 90% organic 7.0–9.0 min, linear gradient of 90–10% organic 9.0–9.1 min, re-equilibrate at 10% organic 9.1–12.0 min). Deconvolution of protein charge ladders was achieved using either Advion Data Express software or the maximum entropy algorithm provided by Analyst 1.4.2 software.

### UV-Vis

Estimates of protein concentration were determined using a Nanodrop^TM^ ND-1000 spectrophotometer (ThermoFisher). Protein concentrations were estimated by absorbance at 280 nm using extinction coefficients calculated from the protein sequence (Expasy ProtParam).

### NMR

Protein NMR samples contained 0.02–0.3 mM of the target proteins suspended in NMR buffer (10% ^2^H_2_O, 20 mM PIPES, pH 6.8, 50 mM NaCl, 0.02% NaN_3_). The PIPES buffer system was selected for its broad useful pH range and for stability of pKa against temperature variation [45]. No correction for the effect of ^2^H_2_O on pH was made. All samples were referenced internally with the water ^1^H resonance relative to DSS in the standard temperature-sensitive way (e.g. 4.78 ppm at 25°C). All ^15^N-HSQC NMR spectra were collected on the following Bruker Avance III HD NMR spectrometers: 11.7 Tesla (^1^H resonance frequency of 500 MHz) unit equipped with a broadband (^1^H-X) SmartProbe or (^1^H/^13^C/^15^N) TXI Probe (Western Washington University, Bellingham, WA, USA); and 14.0 Tesla (^1^H resonance frequency of 600 MHz) instrument equipped with a (^1^H/^13^C/^15^N) TCI Cryoprobe (University of British Columbia, Vancouver, BC, Canada). For the ^15^N-HSQC data, 2048 direct and 256 or 512 indirect points were recorded in the uniform sampling regime unless otherwise noted in the respective figure legends. The ^15^N-HSQC data was recorded at temperature values of 15°C, 25°C and 45°C. For backbone resonance assignment purposes, the following standard 3D heteronuclear data were collected: HNCACB, CBCA(CO)NH, HN(CA)CO, HNCO. The collected data were processed with NMRPipe software (8.9 Rev 2016.307.12.43 32-bit) [46, 47]. The obtained spectra were visualized and analyzed with NMRViewJ software (One Moon Scientific Inc.).

### Analytical ultracentrifugation

The oligomeric state of G_5_-HP60 was examined using analytical ultracentrifugation. All data were collected using a Beckman-Coulter Optima XL-A analytical ultracentrifuge. For sedimentation velocity data, samples were analyzed in standard epon sectored cells at a rotor speed of 42,000 rpm. Sedimentation was monitored using the optical absorbance at 280 nm. For sedimentation equilibrium data, samples were analyzed in 6-channel epon cells at rotor speeds of 28,000, 36,000 and 42,000 rpm. The sample gradient was monitored using the optical absorbance at 284 nm. All sedimentation data were collected at 20°C in 20 mM PIPES, 50 mM NaCl pH 6.8. Sedimentation velocity data were analyzed using the software SEDFIT [48]. Sedimentation equilibrium data were analyzed using the software Heteroanalysis [49].

Analytical ultracentrifugation data are summarized in **S1 Fig**. Sedimentation velocity data confirmed that G_5_-HP60 is a single species in solution with a sedimentation coefficient of ~1 S (**S1A Fig**). This sedimentation coefficient is consistent with the expected molecular weight of the monomeric species of 7491 Da, assuming a frictional coefficient of 1.32. Further analysis of the sample with sedimentation equilibrium supports that the species is a monomer in solution. Initial analysis of the equilibrium data using a single ideal species model yielded a molecular weight somewhat lower than the expected monomer molecular weight, suggesting that either non-ideality or sample degradation may be playing a role. G_5_-HP60 is highly charged, which would likely increase the effect of non-ideal interactions in sedimentation equilibrium, due to charge-charge repulsion between protein molecules. Alternatively, sample degradation could also contribute to a reduction in the observed molecular weight, however, we did not see any evidence of degradation using either SDS-PAGE or mass spectrometry on the samples used for sedimentation equilibrium. Therefore, we further analyzed the data using a non-ideal model in Heteroanalysis, with a fixed molecular weight equal to the monomer. The resulting fits were good with a modest second virial coefficient of 1.4e-5. An example fit of the data is shown in **S1B Fig**. We conducted similar fits with the dimeric molecular weight, however these resulted in larger residuals and a higher second virial coefficient, suggesting that the data did not fit the dimeric model as well as it did the monomeric model. Taken together, the sedimentation velocity and sedimentation equilibrium data confirm that the protein is monomeric in solution.

### Plasmid preparation

The pET-30b(+) expression vector encoding heptamutant sortase A (SrtA_7M_) was obtained from the laboratory of Hidde Ploegh (Addgene plasmid #51141) [50–52]. All remaining expression vectors, which combine the native villin 4 fragments IDR and HP63 with affinity purification tags (His_6_ and FH8) as well as enzyme processing sites (sortase LPXTG motif and TEV cleavage site ENLYFQG), were obtained by commercial gene synthesis. Variants of FH8-IDR, full length FH8-IDR-HP63, and TEV-GG-HP63 were purchased from ATUM and were synthesized with the desired fusion tags and motifs in the pD441-SR plasmid backbone. IDR-HP(877–974) and FH8-EDEED were obtained from Genscript and prepared in the pET-24a(+) plasmid backbone. Full sequences for all proteins used in this study are given in **S2 Fig.**

### General protein expression and purification

Expression vectors were transformed using heat shock into competent *E*. *coli* BL21(DE3) cells, and then the cells were plated on LB agar plates containing kanamycin (10 μg/mL). Single transformants were then grown in LB broth (10 g/L tryptone, 5 g/L yeast extract, 10 g/L NaCl, 10 mg/L kanamycin, pH 7) at 37°C with agitation at 210 rpm. Cells were grown to an optical density of 0.4–0.6 at 600 nm, and then induced with IPTG (0.8 mM final concentration) for 5 h at 37°C. The cells were harvested by centrifugation (6118 x g, 20 min, 4°C), and the cell pellets were then resuspended in lysis buffer (300 mM NaCl, 50 mM NaH_2_PO_4_, 10 mM imidazole, pH 8.0). Lysozyme was then added (50 μg/mL final concentration) and the suspensions were incubated at 4°C for 30 minutes with gentle agitation. The cells were then further lysed by sonication, followed by treatment with DNAse I (0.1 units/mL) for 30 min at room temperature. Cell lysates were then centrifuged (39000 x g, 30 min, 4°C) and the supernatants were passed through a 0.22 μm filter. For proteins containing His_6_ tags, the clarified supernatant was then added to 3–5 mL of immobilized metal affinity chromatography (IMAC) resin (Ni-NTA or Talon® metal affinity resin) and the mixture was incubated for 1 hour at 4°C with gentle agitation. The slurry was then poured into a fritted glass column and the flow through was discarded. The resin was next washed with at least 8 column volumes of wash buffer (300 mM NaCl, 50 mM NaH_2_PO_4_, 20 mM imidazole, pH 8.0). The protein was then eluted with 3 column volumes of elution buffer (300 mM NaCl, 50 mM NaH_2_PO_4,_ 250 mM imidazole, pH 8.0) separated into six fractions. For full length FH8-IDR-HP63, which lacks a His_6_ tag, purification was achieved via hydrophobic interaction chromatography (HIC) relying on the FH8 domain as described by Costa et al. [53]. All proteins underwent a final purification by size exclusion chromatography (SEC) using 20 mM PIPES (pH 6.8), 50 mM NaCl buffer as the eluent.

For the incorporation of ^13^C and/or ^15^N, transformed cells were grown in LB broth (10 g/L tryptone, 5 g/L yeast extract, 10 g/L NaCl, 10 mg/L kanamycin, pH 7) at 37°C to an optical density of 0.4–0.5 at 600 nm. The cells were then collected by centrifugation (4,000 x g, 20 min, 4°C) and resuspended in 0.5 L of salt wash (200 g/L vitamin B1, 120 g/L MgSO_4_, 28 g/L CaCl_2_, 10 mg/L kanamycin). The cells were centrifuged again as previously described, and then suspended in 0.5 L of M9 minimal media (200 g/L vitamin B1, 120 g/L MgSO_4,_ 28 g/L CaCl_2_, 10 mg/L kanamycin, 9 g/L glucose (^13^C-labeled or unlabeled) and 3 g/L ^15^NH_4_Cl. The cells were then grown at 37°C to an optical density of 0.4–0.6 at 600 nm, followed by induction of protein expression with IPTG (0.8 mM final concentration) for 4–5 hours at 37°C. All labeled proteins were purified as described above.

All protein samples were characterized by LC-ESI-MS and/or SDS-PAGE prior to NMR characterization or use in sortase-mediated ligation reactions. (**S3 Fig** and **S1 Table**).

### Preparation of TEV-GG-HP63 and TEV protease cleavage to generate GG-HP63

An identical protocol was employed for the preparation of unlabeled and ^15^N-labeled GG-HP63. First, full length TEV-GG-HP63 (unlabeled or ^15^N-labeled) was expressed and purified following the general protocol described above. Purified TEV-GG-HP63 was then combined with TEV protease in PIPES buffer (20 mM PIPES, 50 mM NaCl, pH 6.8) containing 0.5 mM EDTA and 1 mM DTT at a 100:1 molar ratio of substrate to protease. TEV cleavage reactions were incubated overnight at room temperature, and then the free GG-HP63 protein was separated from unreacted TEV-GG-HP63 and His_6_-tagged TEV protease using IMAC. Briefly, TEV reaction mixtures were combined with Ni-NTA slurry (volume of Ni-NTA used was generally ~10–20% (v/v) of the original TEV-cleavage reaction volume). This mixture was transferred to a fritted gravity flow column, and then capped and left to equilibrate with gentle agitation at 4°C for 1 h. The flow through containing free GG-HP63 was then collected, and the resin was washed with additional buffer (20 mM PIPES, 50 mM NaCl, pH 6.8) to collect residual GG-HP63. If necessary, the free GG-HP63 headpiece was further purified by SEC. For all preparations, the identity and purity of GG-HP63 (both unlabeled or ^15^N-labeled) was confirmed by SDS-PAGE and LC-ESI-MS (**S3 Fig** and **S1 Table**).

### Preparation of ^15^N-labeled FH8 domain via TEV protease cleavage

Purified FH8-IDR-HP63 (uniformly ^15^N-labeled) was combined with TEV protease in PIPES buffer (20 mM PIPES, 50 mM NaCl, pH 6.8) containing 0.5 mM EDTA and 1 mM DTT at a 50:1 molar ratio of substrate to protease. The total reaction volume was 1 mL. The reaction was incubated overnight at room temperature, and then free FH8 was isolated by hydrophobic interaction chromatography (HIC) using Phenyl Sepharose^TM^ 6 Fast Flow (high sub) resin (GE Healthcare). Briefly, the crude reaction was treated with 300 μL of HIC resin that had been equilibrated with binding buffer (50 mM Tris, 150 mM NaCl, 5 mM CaCl_2_, pH 7.6). The mixture was incubated for 1 h, and then the resin was washed three times with 600 μL of wash buffer (25 mM Tris, 75 mM NaCl, 2.5 mM CaCl_2_, pH 7.6). The protein was eluted off with 300 μL of elution buffer (50 mM Tris, 5 mM EDTA, 150 mM NaCl, pH 7.6). Cleaved FH8 (^15^N-labeled) was then further purified by SEC, and its identity and purity were confirmed by LC-ESI-MS and SDS-PAGE (**S3 Fig** and **S1 Table**).

### Preparation of ^15^N-labeled G_5_-HP60 via sortase-mediated cleavage

A 200 μL reaction containing uniformly ^15^N-labeled IDR-G_5_-HP60 (200 μM final concentration), SrtA_7M_ (50 μM), and GG dipeptide (2 mM) in PIPES buffer (20 mM PIPES, 50 mM NaCl, pH 6.8) was incubated overnight at 37°C. The crude reaction was then treated with 200 μL of Ni-NTA slurry and gently mixed for 2 h at room temperature. The mixture was then filtered and the flow through containing G_5_-HP60 was collected. The purity of ^15^N-labeled G_5_-HP60 was confirmed by SDS-PAGE (**S3 Fig**).

### Model sortase-mediated ligations between FH8 substrates and triglcyine (GGG)

Model ligations were performed on 100 μL scale. Stock solutions of all reaction components were prepared in PIPES buffer (20 mM PIPES, 50 mM NaCl, pH 6.8) and combined in appropriate ratios to give the desired reagent concentrations. In all cases, final concentrations of 50 μM FH8/FH8-IDR substrate, 10 mM triglycine (GGG), and 10 μM SrtA_7M_ were used. Reactions were incubated at room temperature and analyzed at 30 minute time intervals by LC-ESI-MS. Reaction progress was estimated from mass spectra by comparing the deconvoluted peak areas of the unmodified FH8/FH8-IDR substrate and the corresponding ligation product. In addition to the expected peaks for the substrate and ligation product, mass spectra consistently showed the appearance of adducts corresponding to the addition of ~41 Da. These can be attributed to the formation of MeCN adducts arising from the LC-ESI-MS mobile phase. Peak areas for these adducts were included in the overall amount of substrate or ligation product for the estimation of reaction conversion.

### Preparation of segmentally labeled FH8-IDR-HP63 and IDR-HP63

Sortase-mediated ligations between unlabeled or ^15^N-labeled samples of FH8-IDR-G and GG-HP63 were conducted on ~1.0–3.5 mL scale. All reactions were run in PIPES buffer (20 mM PIPES, 50 mM NaCl, pH 6.8) with final reagent concentrations of 100 μM FH8-IDR-G, 50 μM GG-HP63, and 10 μM SrtA_7M_. Reactions were incubated at room temperature for 1–4 h. The desired segmentally labeled derivatives were then separated from unreacted FH8-IDR-G and His_6_-tagged SrtA_7M_ using IMAC. Briefly, crude reaction mixtures were combined with Ni-NTA slurry (volume of Ni-NTA used was generally 50% (v/v) of the original ligation reaction volume). This material was transferred to a fritted gravity flow column, then capped and left to equilibrate with gentle agitation at 4°C for 1 hour. The flow through containing the segmentally labeled protein was then collected, and the resin was washed with at least three additional resin volumes of buffer (20 mM PIPES, 50 mM NaCl, pH 6.8) to collect residual product. All segmentally labeled products were then further purified by SEC. The identity and purity of all ligation products were assessed by SDS-PAGE and LC-ESI-MS. An identical protocol was used to prepare FH8-IDR-HP63 segmentally labeled with ^13^C and ^15^N in the FH8-IDR segment.

For removal of the FH8 tag, purified FH8-IDR-HP63 (segmentally labeled with ^15^N in the FH8-IDR portion) was combined with TEV protease in PIPES buffer (20 mM PIPES, 50 mM NaCl, pH 6.8) containing 0.5 mM EDTA and 1 mM DTT at a 50:1 molar ratio of substrate to protease. The reaction was incubated overnight at 4°C. The cleaved FH8 domain and His_6_-tagged TEV protease where then removed from the reaction mixture using HIC and IMAC, respectively, according to the protocols described above. Free IDR-HP63 was then buffer exchanged into PIPES buffer (20 mM PIPES, 50 mM NaCl, pH 6.8) using an Econo-Pac 10DG desalting column (Bio-Rad) following the manufacturer’s instructions. This material was further concentrated using an Amicon® Ultra-15 centrifugal filter (3 kDa NMWL) and the identity and purity of free IDR-HP63 were then confirmed by LC-ESI-MS and SDS-PAGE.

## Results and discussion

**Fig 2:**
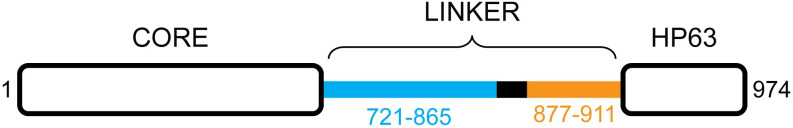
Schematic representation of villin 4. The N-terminal core fragment and C-terminal headpiece domain (HP63) are joined by a mostly disordered linker (positions 721–911). The linker has N-terminal basic (pI 11.5, shown in blue) and C-terminal acidic (pI 4.1, shown in orange) regions. A predicted 11-residue PEST motif (shown in black) separates the basic and acidic regions of the linker.

Native villin 4 contains a 190-residue fragment (positions 721–911) that is predicted to be mostly disordered [54]. Further analysis of this IDR reveals that it consists of a basic stretch at its N-terminus (~145 residues), followed by a predicted regulatory PEST motif [55, 56] and a C-terminal acidic portion (35 residues) (**Figs 2** and **S4**). As an extension of our previous work with the villin 4 headpiece domain in isolation [54], we elected to focus our segmental isotopic labeling studies on a construct consisting of the 35-residue acidic IDR fragment joined to the headpiece. To render this system compatible with SML, it was first necessary to construct an IDR derivative fused at its C-terminus to an LPXTG sortase recognition motif, along with a headpiece derivative displaying an accessible polyglycine unit at its N-terminus (**Fig 1**). In order to facilitate purification and enhance the stability of IDR-containing samples against proteolysis, a folded 71-residue FH8 domain tag was inserted at the N-terminus of the IDR fragment (**S2 Fig**) [53]. To allow removal of this FH8 tag, a TEV protease cleavage site was inserted in between FH8 and the IDR as well.

### Sortase-mediated ligation for assembly of segmentally labeled villin 4 constructs

**Fig 3:**
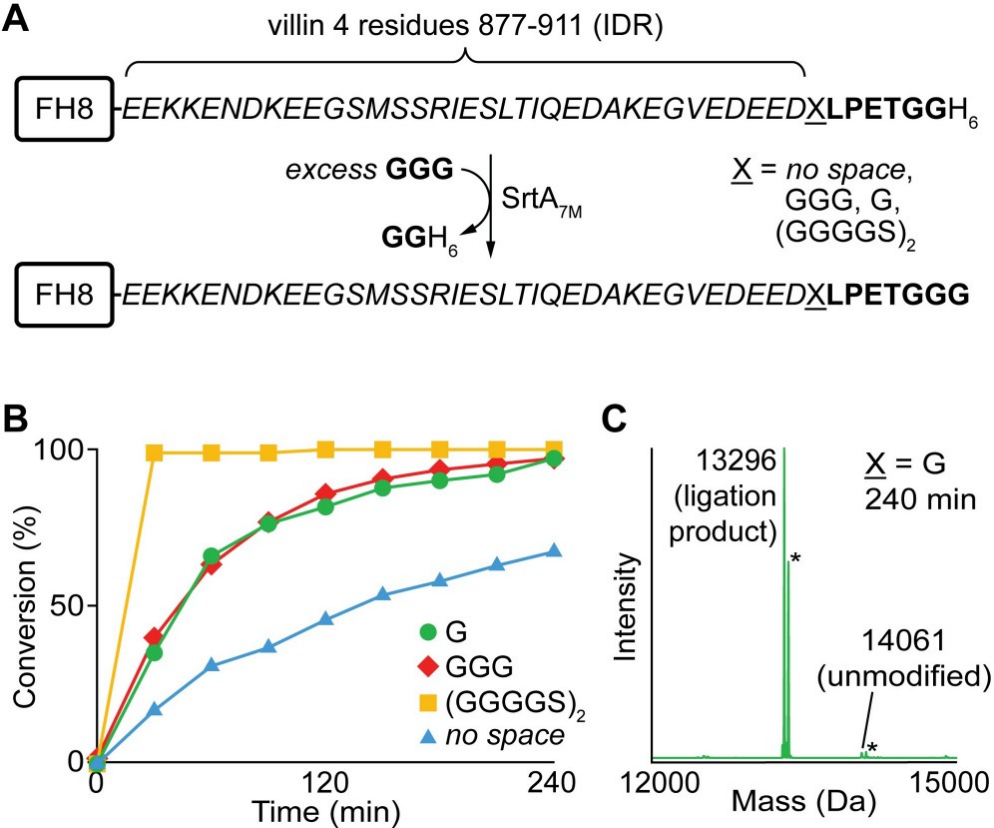
Effect of linker structure on SML using villin 4 IDR substrates. (**A**) Model SML reaction for comparing ligation efficiency with IDR substrates containing different spacers. (**B**) Time course demonstrating improved ligation efficiency when spacers were included between the IDR and LPXTG motif. Reaction progress was estimated using LC-ESI-MS. (**C**) ESI-MS spectrum of SML reaction mixture for the substrate containing a single glycine (G) spacer following a 4 h incubation at room temperature (calculated MWs: unmodified substrate = 14062 Da, ligation product = 13296 Da, * = MeCN adducts from LC-ESI-MS mobile phase).

Beginning with the IDR fragment, we initially expressed a variant with the IDR residues directly fused to an LPETGG motif. The additional glycine following the LPXTG site was included as it is known to improve SML reaction rates *in vitro* [57]. An FH8 domain and His_6_ affinity purification handles were also included at the N- and C-termini of the IDR construct, respectively (**Figs 3A** and **S2**) [53]. To assess recognition of this substrate, a model reaction was performed in which it was ligated to an excess of a simple triglycine peptide in the presence of a catalytically enhanced version of SrtA possessing seven point mutations (SrtA_7M_) (**Fig 3A**) [50–52]. While LC-ESI-MS revealed the formation of the desired ligation product, overall rates were modest and the reaction failed to reach completion during a 4 hour incubation period at room temperature (**Figs 3B** and **S5**).

While analyzing the efficiency of the SML reaction, we noted that the LPETGG site in our initial design was adjacent to a highly acidic pentapeptide at the very C-terminus of the villin 4 IDR fragment (E_907_DEED_911_). Given that the L residue of the LPXTG motif sits within a hydrophobic pocket in the sortase substrate binding site [58, 59], we hypothesized that this hydrophilic, acidic pentapeptide may be hindering substrate recognition, leading to slow and incomplete ligation. To address this, we prepared a series of derivatives with spacers of various size placed between the EDEED sequence and the inserted LPETGG motif (**Fig 3A**). In model reactions, the substrate with the largest spacer (GGGGS)_2_ demonstrated the highest reaction rates, with complete ligation in under 30 minutes. Two shorter spacers (GGG or G) resulted in acceptably fast reactions in which reactions reached >90% completion within four hours. In all cases, the formation of the desired ligation products was confirmed by LC-ESI-MS (**Figs 3C** and **S5**). Overall, these observations supported our hypothesis that the acidic pentapeptide (EDEED) was slowing the SML process. As further evidence for this phenomenon, we also observed relatively slow ligation reactivity for a model FH8 substrate in which the EDEED residues alone were positioned directly N-terminal to the LPETGG motif (**S6 Fig**). Importantly, all of these studies also revealed that the detrimental effect of the EDEED sequence could be minimized by the inclusion of a single glycine spacer, which was then utilized in all subsequent ligation reactions to maintain suitable reaction rates with minimal perturbation of the native IDR sequence.

**Fig 4:**
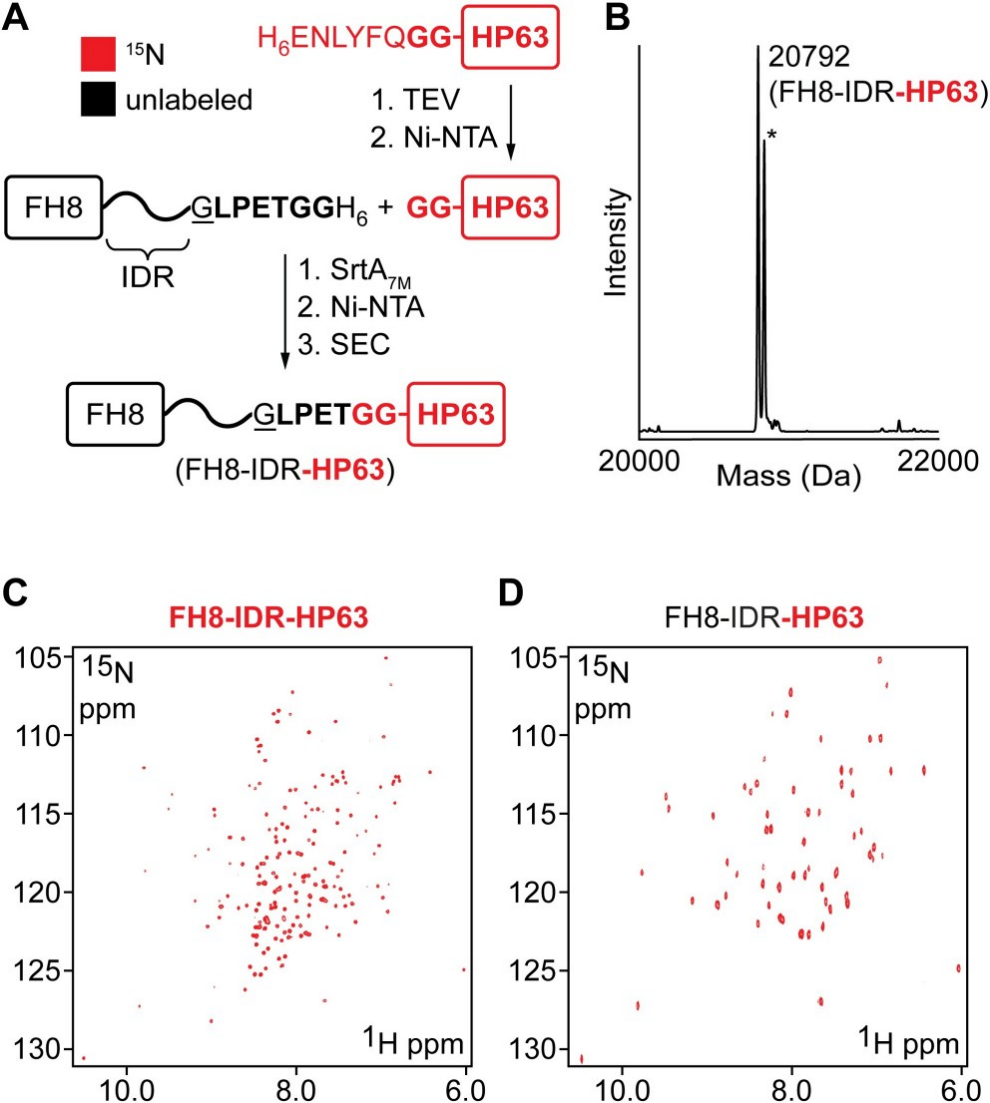
Segmental isotopic labeling of truncated villin 4. (**A**) SML strategy for producing segmentally labeled derivatives containing an IDR segment (35-residue acidic fragment) and the headpiece domain of villin 4. (**B**) ESI-MS spectrum of purified FH8-IDR-HP63 with selective incorporation of ^15^N in the HP63 segment (calculated MW assuming 100% ^15^N incorporation in HP63 = 20797 Da, * = MeCN adducts from LC-ESI-MS mobile phase). (**C**) ^15^N-HSQC (500 MHz, 25°C) of uniformly labeled FH8-IDR-HP63. Based on the sequence of FH8-IDR-HP63, 176 resonances originating from non-proline residues are expected and 173 were observed. (**D**) ^15^N-HSQC (500 MHz, 25°C) of segmentally labeled FH8-IDR-HP63 in which ^15^N labeling is restricted to HP63. Based on the sequence of the labeled segment, 61 resonances originating from non-proline residues are expected and 58 were observed.

For the production of the C-terminal ligation partner, we utilized a 63-residue headpiece fragment (HP63, residues 912–974 in villin 4) that was previously shown to adopt a stable fold in solution [54]. For use in SML, we further augmented the HP63 polypeptide with an N-terminal diglycine that could be revealed upon TEV protease cleavage of a His_6_ affinity tag. This construct was generated in ^15^N-labeled form, and then used to prepare a segmentally labeled derivative as outlined in **Fig 4A**. As in the case of the model IDR substrate reactions (**Fig 3**), SrtA_7M_ was able to efficiently ligate the labeled and unlabeled fragments, yielding the desired segmentally labeled product in which ^15^N was selectively incorporated into the HP63 portion. For purification, the ligation reaction mixture was passed over Ni-NTA resin to remove His_6_-tagged SrtA_7M_ and any unreacted IDR substrate. The eluent was further purified using SEC to separate the ligation product from free headpiece. Following these steps, pure segmentally labeled product (FH8-IDR-HP63) was isolated and its identity and purity were confirmed by SDS-PAGE and LC-ESI-MS. (**Figs 4B** and **S7A**).

### NMR analysis of segmentally labeled constructs

The construct consisting of unlabeled FH8-IDR and ^15^N-labeled headpiece reported a ^15^N-HSQC signature nearly identical to that of the isolated headpiece domain (**Figs 4D** and **S8**) [54]. The same headpiece signature was also present in the ^15^N-HSQC spectrum of a fully ^15^N-labeled analog (**Fig 4C**), however as expected several of the resonances were obscured by overlap with signals from the IDR. As a final comparison, we also acquired the ^15^N-HSQC spectrum of a native 98-mer (IDR-HP(877–974), residues 877–974 in villin 4) consisting of the 35-residue acidic IDR linker fragment directly fused with HP63, thus omitting the sortase ligation site (**S2** and **S9 Figs**). From this, we determined that the observed headpiece signatures in the segmentally labeled product were nearly identical to the headpiece resonances of the native C-terminal 98-mer. These observations confirmed that the headpiece domain in the SML product retained its characteristic fold.

**Fig 5:**
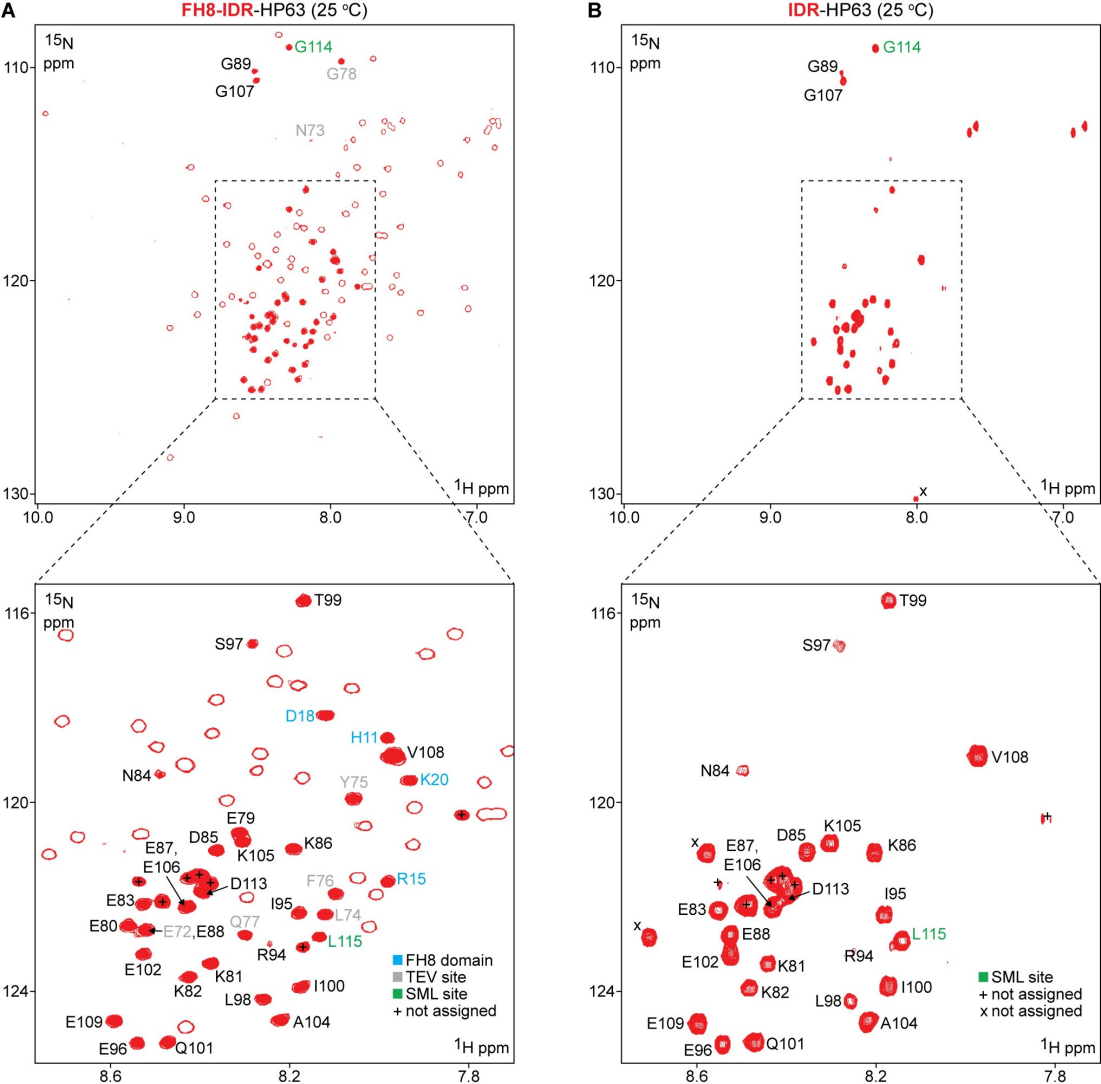
^15^N-HSQC NMR spectra (25°C) of segmentally labeled FH8-IDR-HP63 and IDR-HP63. **(A)** Spectrum recorded at 600 MHz (^1^H frequency) with 256 indirect increments using a FH8-IDR-HP63 sample with the FH8-IDR portion segmentally labeled with ^13^C/^15^N isotopes. All observed resonances for the labeled FH8-IDR segment are represented by open red contours, and signals that have been segmentally assigned to the IDR linker portion are overlaid with solid red ovals. Residue-specific NMR assignments for 36 residues in the linker region (27 from the villin 4 IDR and 9 residues from the flanking TEV and sortase ligation sites) and four FH8 domain peaks (false positives from the segmental assignment) are indicated with residue numbers. The numbers for the TEV site (7 residues) are shown in grey, FH8 domain (4 residues) are shown in blue, and two residues corresponding to the SML ligation site (G114, L115) are shown in green. Seven segmentally assigned peaks for which no residue specific assignments were obtained are marked with “+”. Residue numbers shown correspond to the sequence of FH8-IDR-HP63 (see **S2 Fig**). To relate the assigned IDR residues to the corresponding positions in wild type villin 4, a value of 798 should be added. (**B**) Spectrum recorded at 500 MHz (^1^H frequency) with 196 indirect increments using an IDR-HP63 sample with the IDR segmentally labeled with ^15^N isotopes. All observed resonances for the labeled IDR segment are represented by solid red signals. Residue-specific NMR assignments for 25 residues in the linker region were unambiguously transferred from panel **A** (out of 27 listed on panel **A**). Three new resonances were observed (marked with “x”) following removal of FH8. Two of these are presumed to correspond to residues E79 and E80, which are adjacent to the newly formed N-terminal residue G78. The two residues corresponding to the SML ligation site (G114, L115) are shown in green. As in the case of FH8-IDR-HP63 (panel **A**), additional peaks (6 total) for which no residue specific assignments were obtained are marked with “+”.

We next generated the complementary segmentally labeled construct (FH8-IDR-HP63) in which the N-terminal fragment (FH8-IDR) was ^13^C/^15^N-labeled while the headpiece remained unlabeled (**Fig 5**). As expected, the corresponding ^15^N-HSQC spectra reported the signatures of the FH8 domain, as determined by comparison with the ^15^N-HSQC of free FH8 (**S10A Fig**). Importantly, the correspondence between free FH8 signals and those of the segmentally labeled construct confirmed that the FH8 tag folds and acts independently from IDR-HP63 in the FH8-IDR-HP63 fusion. The pattern of resonances observed in the ^15^N-HSQC spectrum of FH8 in isolation allowed us to identify the majority of the FH8-specific signals in the spectrum of the segmentally labeled FH8-IDR-HP63. These signals could therefore be excluded, leaving the remaining resonances as likely originating from the IDR. By treating these putative IDR peaks as a collective, and without residue-specific assignment of individual resonances, we were thus able to complete what we term the segmental assignment of the IDR (**Fig 5A**). We note here, that there is the potential for signal overlap and chemical shift differences between FH8 in isolation versus FH8 fused to the IDR-HP63 fragment. As a result, a certain number of false positives (non-IDR peaks assigned to the IDR) were expected from segmental assignment. However, the frequency of these was anticipated to be low due to the modularity of the FH8-IDR-HP63 system.

The segmental assignment procedure described above yielded 40–45 peaks, with minor uncertainty in the overall peak count due to resonance overlap in the IDR chemical shift range (**Fig 5A**). Reassuringly, this procedure revealed only resonances within the characteristic ^1^H chemical shift range of IDRs (7.8–8.7 ppm) [60], suggesting that they may belong to unstructured protein regions. Based on the sequence and isotopic labeling pattern of the FH8-IDR-HP63 construct, the segmental assignment procedure was anticipated to reveal 40 resonances. Of those, 35 should correspond to the acidic IDR fragment, four were expected from the GLPET residues installed at the C-terminal sortase ligation site, and one additional signal should arise from the G at the P_1_’ position of the N-terminal TEV site inserted between the FH8 and IDR regions. Thus, the overall agreement between the number of resonances expected and the number of resonances identified via segmental assignment was very good.

In order to assess the accuracy of the segmental assignment, we next employed a minimal, standard suite of 2D/3D heteronuclear NMR experiments (^15^N-HSQC, HNCACB, CBCA(CO)NH, HNCO, HN(CA)CO) to perform the residue-specific backbone resonance assignment of the FH8-IDR portion of the ligation product segmentally labeled with ^13^C/^15^N isotopes. Importantly, segmental labeling greatly simplified the residue-specific assignment process, as all resonances from the HP63 domain were absent in the spectra. With the newly acquired 3D heteronuclear data, we were able to unambiguously assign 97 out of 115 non-proline residues (**Fig 5A**, BMRB entry: 50205). Within the 35-residue acidic IDR fragment, 27 (77%) residues were assigned. Seven out of the eight unassigned residues occupy two segments in the sequence: positions 90–93 (three Ser residues out of four in the segment) and 110–112 (all are Glu and Asp) (**S2 Table**). Most likely, a significant fraction of the unassigned resonances cluster within a massive overlap located near 8.40 ppm (^1^H) / 121.5 ppm (^15^N). It is also possible that the unassigned segments display intermediate time scales of conformational motion and/or exchange with the solvent and thus were not detected on the spectra. Residue-specific assignment also revealed that four of the segmentally assigned peaks (H11, R15, D18, K20; **Fig 5A**) originated from FH8. This confirmed our expectation of a low level of false positives obtained from the segmental assignment strategy. Additionally, the resonances indicated with only open red contours in **Fig 5A** were assigned exclusively to the FH8 sequence, which further supported the accuracy of our segmental assignment approach. We also note that no spectroscopic indications of a formally possible *cis-trans* isomerization of the proline introduced within the sortase ligation site were detected.

As another test of the validity of our segmental assignment, we also utilized the aforementiond IDR-HP(877–974) control sample, which contains native villin 4 residues 877–974 and lacks both the SML and TEV cleavage sites of the segmentally labeled derivative. Of the 40–45 resonances revealed by segmental assignment of FH8-IDR-HP63 (**Fig 5A**), 26 had indisputable counterparts in the native control spectrum, and 4 had likely matches with chemical shift differences of <0.05 ppm (^1^H axis) and <0.2 ppm (^15^N axis) (**S11 Fig and S2 Table**). With respect to the 27 IDR residues identified via residue-specific assignment, all were found to have counterparts in the spectrum of the IDR-HP(877–974) native control (**S11 Fig and S2 Table**). Taken together, this suggested that insertion of residues for sortase-mediated ligation (GLPETGG) between the IDR linker and headpiece had minimal perturbations on a major portion of the IDR. That said, we acknowledge that the insertion of 5–7 additional residues (e.g. LPXTG or GLPETGG) for sortase-mediated ligation may not be universally tolerated in all IDRs without compromising functional properties for some samples. For those cases, we propose instead considering suitable point mutations for function-neutral conversion of a native sequence to an LPXTG sortase substrate site. In addition, while not explored here, functional tests of the modified polypeptides are suggested to determine the extent to which sequence modifications alter the key functional parameters of the protein samples.

Since the FH8 tag represented a sizable protein domain, we next sought to determine whether its presence impacted the observed spectral signatures of the IDR residues. Starting from our segmentally labeled FH8-IDR-HP63 construct, we proceeded to cleave the FH8 tag using TEV protease. This generated a segmentally labeled sample we termed IDR-HP63 (**S2 and S7C Figs**), with ^15^N-labeling restricted to the IDR segment. In this sample the FH8 tag is absent, while the GLPETGG sortase ligation site connecting the ^15^N-labeled IDR and unlabeled headpiece (HP63) domain is retained. To elucidate the effects of the FH8 tag, we compared the ^15^N-HSQC spectrum for IDR-HP63 with the analogous spectrum for the segmentally-labeled FH8-IDR-HP63 sample (**Fig 5A and 5B**). With the exception of a few N-terminal residues, resonances for all the assigned IDR residues were found at nearly identical positions for FH8-IDR-HP63 and its FH8-free counterpart IDR-HP63 (changes <0.05 ppm for ^1^H and <0.2 ppm for ^15^N values, **S2 Table**). In terms of differences, only the two N-terminal IDR residues that were initially adjacent to the FH8/TEV portion (E79, E80) demonstrated noticeable ^15^N-HSQC perturbation, specifically peaks repositioning and/or disappearing. While it is possible that signals from these residues are absent or obscured by other resonances, we consider it likely that they correspond to two of the newly observed signals unique to IDR-HP63 (indicated with “x” in **Figs 5B** and **S13**). Resonances for the next two residues (K81, K82) moved, but remained nearby and their pattern was straightforward to identify. The comparison of the ^15^N-HSQC signatures of the FH8-free variant (IDR-HP63, **Fig 5B**) and our native control (IDR-HP(877–974) (**S9 Fig**) also provided additional evidence that the GLPETGG inserted for SML had virtually no effect on the included portion of the native IDR (**S2 Table**). Only two residues (K81 and K82) exhibited any appreciable effects, which can be explained by the fact that the two samples compared had chemical differences at their N-termini: Gly78 for IDR-HP63 (the single remaining residue from the TEV cleavage site) and His_6_ for IDR-HP(877–974).

### Probing temperature sensitivity in the villin 4 IDR using NMR

The success of the segmental labeling and assignment procedure prompted us to consider whether this strategy could provide a rapid, preliminary assessment tool for relevant IDR properties. In the present villin 4 fragment, we were particularly interested in evaluating temperature sensitivity of the 35-residue segment of the IDR. Our interest stemmed from the fact that many plants operate within a relatively broad temperature range (from subzero to more than 40°C). Thus, understanding modulation of protein properties in response to temperature variation is central for plant biochemical and physiological studies.

In regards to plant villin 4, the N-terminal subdomain of the headpiece (residues 912–940, **S4 Fig**) was previously shown to be temperature sensitive and exhibited significant tertiary structure alterations over the range of 10–40°C [54]. In addition, replacement of the native E_910_DLPA_914_ residues on the surface of the N-terminal subdomain with GGGGG resulted in major destabilization of the headpiece when this fragment (G_5_-HP60, **S2 Fig**) was characterized in isolation. Specifically, the ^15^N-HSQC spectrum of free G_5_-HP60 at 25°C appeared markedly different from the typical villin 4 headpiece pattern, exhibiting only 20 of the more than 50 signals expected (**S12 Fig**). Remarkably, for the G_5_-HP60 peaks detected, nearly all coincided with previously observed signals for the highly stable 34-residue C-terminal headpiece subdomain (villin 4 residues 941–974) [54]. No peaks corresponding to the less stable 29-residue N-terminal subdomain (villin 4 residues 912–940) were identified on the G_5_-HP60 ^15^N-HSQC spectrum recorded at 25°C [54]. Sedimentation velocity and sedimentation equilibrium analytical ultracentrifugation showed that G_5_-HP60 is a single monomeric species in solution, which suggested intermediate time scale dynamics as the reason for peak disappearance in the N-terminal subdomain in G_5_-HP60 at 25°C (**S1 Fig**) [54]. It is worth noting that lowering the temperature to 5°C resulted in restoration of the headpiece-like ^15^N-HSQC pattern of G_5_-HP60 (**S12 Fig**). Importantly, the G_5_-HP60 peaks detected (42 total) at 5°C closely match headpiece resonances from both the more stable C-terminal and more labile N-terminal subdomains (**S12 Fig**). Taken together, these data indicate that G_5_-HP60 is dynamically less stable than the native headpiece. Surprisingly, it takes just a three-residue replacement (L_912_P_913_A_914_ to G_912_G_913_G_914_) at the N-terminus of the headpiece to destabilize the entire N-terminal subdomain, revealing its overall marginal stability. Overall, these data indicate significant temperature sensitivity and low thermodynamic stability of the N-terminal headpiece subdomain.

**Fig 6:**
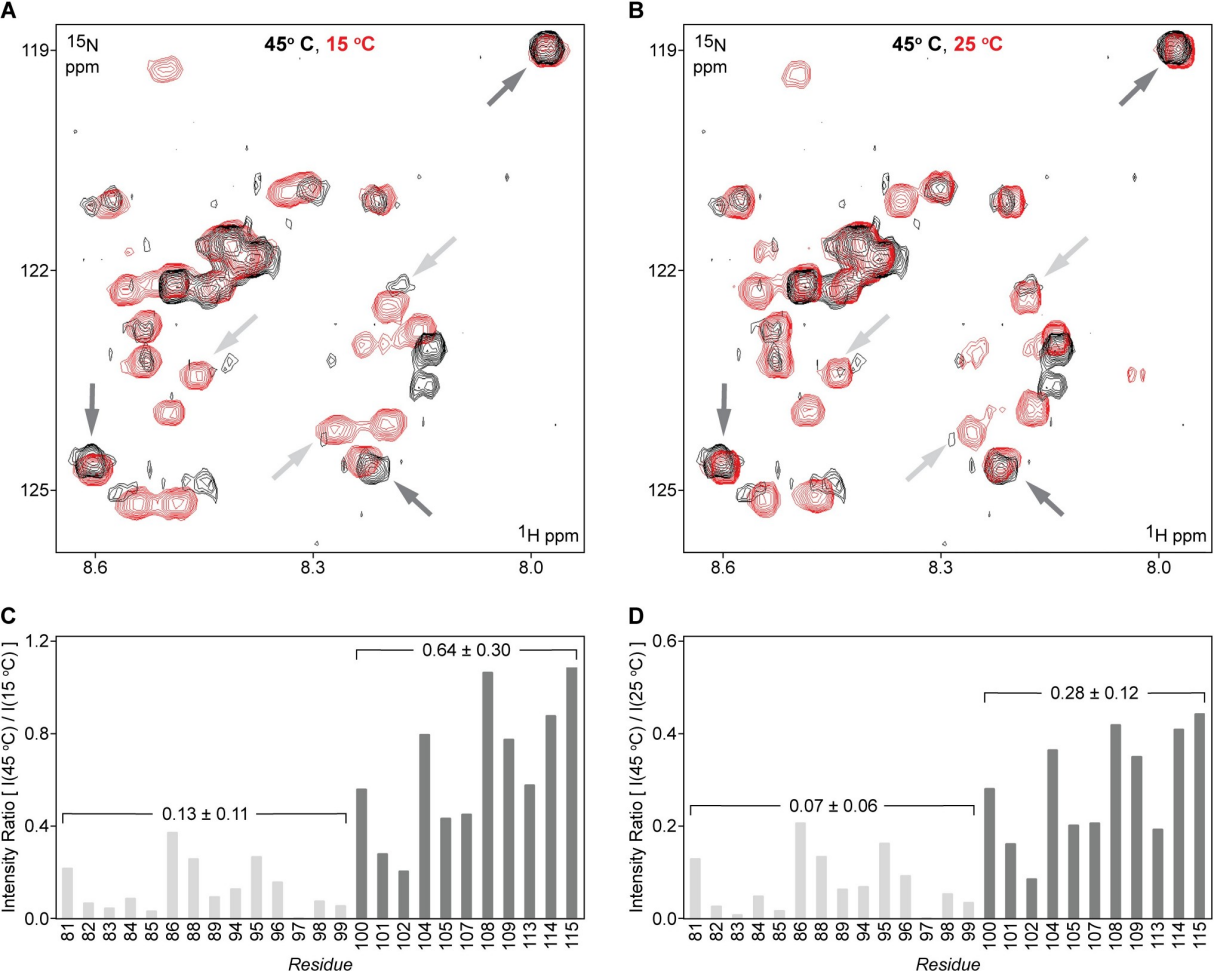
Temperature sensitivity in the 35-residue acidic segment of the villin 4 IDR. Representative regions of ^15^N-HSQC NMR spectra (500 MHz, ^1^H frequency) for IDR-HP63 with the IDR segmentally labeled with ^15^N isotopes recorded at (**A**) 45°C (black) / 15°C (red) and (**B**) 45°C (black) / 25°C (red). Spectra recorded at 15°C and 45°C were re-referenced to the ^1^H / H_2_O chemical shift value (4.78 ppm) of the spectrum recorded at 25°C according to a standard approach [63]. The number of scans for the three spectra were 64 (15°C), 128 (25°C), and 192 (45°C), with 196 indirect increments (^15^N dimension) in all three cases. (**C**) Ratios of ^15^N-HSQC backbone amide signal intensity for assigned IDR residues at 45°C and 15°C, and (**D**) a corresponding data set for signal intensity ratios at 45°C and 25°C. In both panels **C** and **D**, data points for residues 81–99 are colored in light grey, and dark grey for residues 100–115. Light grey and dark grey arrows on panels **A** and **B** point to select signals representing each of these two respective groups. Data points for residues 87 (from the light grey group) and 106 (dark grey group) are excluded from this analysis due to their resonance overlap. Values shown above the brackets represent the average intensity ratio (± standard deviation) for the residues indicated.

Given the higher temperature sensitivity of the headpiece N-terminal subdomain adjacent to the IDR linker, we reasoned that a differential temperature sensitivity may extend to the IDR linker itself. To test this, we utilized the segmentally labeled IDR-HP63 construct (^15^N-labeling in the IDR segment) described above. ^15^N-HSQC spectra of this sample were collected at temperatures of 15°C, 25°C, and 45°C (**Figs 6 and S13**). It is noteworthy that the number of resonances observed at 15°C (39–42) corresponds well to the number of signals expected for the labeled segment of IDR-HP63 (40 non-proline residues). The spectrum recorded at 25°C displayed four fewer peaks than the spectrum obtained at 15°C, possibly due to intermediate timescale of conformational or chemical exchange processes. Inspection of the segmentally assigned IDR resonances recorded at 45°C revealed that approximately 50% of these peaks have highly similar positions and form similar multi-peak patterns to those observed at 15°C and 25°C (**S13 Fig**). The remaining peaks in the 45°C spectrum had dramatically attenuated intensities, suggesting that the acidic part of the IDR linker is sensitive to temperature with the effect being non-uniform throughout the sequence.

The qualitative observation of attenuated intensities for certain peaks at 45°C derived from segmental assignment prompted us to evaluate the temperature sensitivity of the 35-residue segment of the IDR in a residue-specific way. The observed overlap in the signals from the 15°C and 45°C spectra with the signals in the 25°C spectra allowed for the reliable transfer of nearly all of the resonance assignments provided in **Fig 5A** (see also **S13 Fig**). Our examination of relative peak intensities as a function of temperature are quantified in **Fig 6C** and **6D**, which shows the ratio of the ^15^N-HSQC signal intensities recorded at 45°C over the intensity of the same resonances recorded at 15°C or 25°C for each residue number. This analysis revealed a clear pattern; in comparing signal intensities at 45°C and 15°C, the ratio values for assigned residues between positions 81 and 99 (average ± standard deviation = 0.13 ± 0.11) were significantly lower than the ratios obtained for the remainder of the IDR (residues 100–115, average ± standard deviation = 0.64 ± 0.30) (**Fig 6C**). The comparison between 45°C and 25°C yielded a similar pattern (**Fig 6D**); values for positions 81–99 (0.07 ± 0.06) were markedly lower than those for IDR residues 100–115 (0.28 ± 0.12). This effect of temperature on the NMR signal intensity ratios in the two IDR segments (positions 81–99 vs 100–115) suggested a distinction between the segments in terms of their effective tumbling rates and/or conformational/chemical exchange timescales [61, 62]. Both tumbling and exchange regimes within the two segments of the IDR can be differentially influenced by the folded headpiece domain and its more labile N-terminal subdomain, which connects to the IDR at sequence position 115. This segmental temperature effect observed in the IDR also represents a novel example of segmental temperature-induced phenomena in addition to the behavior described above for the adjacent N-terminal and C-terminal subdomains of the native villin-4 headpiece and its G_5_-HP60 derivative. Thus, our data suggest that detailed studies of the temperature dependance of the conformational dynamics of the full-length IDRs (130–190 residues in length) and adjacent domains in plant villins is warranted. Based on our data here, we expect that probing by dedicated NMR experiments (R1/R2, CPMG, CEST) and molecular dynamics simulations will likely reveal other examples of segmental dynamics within these IDRs, which can be potentially relevant for plant protein regulation by temperature. Overall, these outcomes also support the use of segmental assignment for fast initial assessment of target dynamic properties probed on a segment-by-segment basis. This in turn can inform whether dramatically more time-intensive NMR recordings needed for residue-specific assignment and describing functional phenomena of interest (e.g. segmental motion, binding interfaces, etc.) in a residue-specific way are warranted for each particular segment.

## Conclusion

In summary, we have leveraged the capabilities of sortase-mediated ligation (SML) as an emerging method for characterizing sizable intrinsically disordered regions. The success of this strategy necessitated tuning of the environment of the SML site to ensure robust and reliable ligation. Notably, this included the finding that oligopeptides directly N-terminal to the LPXTG site can have a significant impact on the success of the ensuing ligation in terms of reaction rates and yield. While the identity of residues C-terminal to the LPXTG motif has been shown to affect SML reaction rates [57], to our knowledge the role of residues N-terminal to the ligation site has yet to be established. While not explored here, a more systematic and comprehensive assessment of neighboring residue effects in SML is warranted, and would provide valuable guidelines for applying this ligation strategy to other IDR systems.

Within the context of this work, our SML strategy was used to generate derivatives of the plant cytoskeleton regulating protein villin 4 that were segmentally labeled with ^15^N and ^13^C/^15^N isotopes for NMR characterization. Selective incorporation of isotopes into the 35-residue acidic segment of the IDR allowed us to reliably identify the IDR backbone resonances via a process we have termed segmental assignment. In this approach, we relate a group of NMR resonances to a specific selectively labeled segment within an otherwise unlabeled protein context. The observed resonances were then catalogued and analyzed for features of interest as a collective. The robustness of this approach was assessed by a residue-specific assignment of the target segment, which we were able to perform with a minimal set of heteronuclear 3D NMR data. Furthermore, comparison of related constructs possessing or lacking certain non-native features (sortase ligation site, FH8 tag, TEV protease site) revealed only minimal perturbations of the spectral signature of the target IDR.

As a demonstration of the utility of our SML/segmental assignment approach for the characterization of IDRs, we probed the acidic 35-residue C-terminal portion of the IDR linker in plant cytoskeleton regulator villin 4 for temperature sensitivity. This revealed that the C-terminal portion (residues 877–911) of the villin 4 IDR linker has different temperature sensitivity regimes within the labeled fragment. This justified a residue-specific analysis, which identified two segments (residues 879–894 and 895–911) that responded to temperature differently. While these findings were derived from a truncated version (35 residues) of the native plant villin 4 IDR linker, they nonetheless suggest that the full-size linker (190 residues) may possess segments with distinct structural and dynamic properties, as has been shown in the case of other IDRs [64].

Based on our work here with the villin 4 system, we propose that segmental assignment offers significant practical advantages for the analysis of sizable IDRs which complement existing approaches.

For example, a typical workflow for proteins with IDRs includes acquisition of an extensive set of 3D/4D heteronuclear NMR data, with larger IDR samples often necessitating the use of more sophisticated and demanding NMR experiments [19–21]. In those cases, the combined recording times for such analyses may surpass the stability limits of the IDR sample. Moreover, high magnetic field instrumentation is often required for recording such data. The segmental assignment methodology described here relies on expedient 2D NMR recordings as a preliminary assessment of IDR properties, which may help identify shorter segments of particular interest. This in turn provides guidance for segments in which residue-specific analysis is justified. For the shorter segments identified in this fashion, basic heteronuclear 3D NMR involving a smaller number of experiments can be sufficient for residue-specific assignment and analysis, which can also reduce NMR acquisition times substantially. In the present work, both of these advantages were realized. Specifically, the ^15^N-HSQC spectra utilized for segmental assignment were generally recorded in under 2 hours on a basic 500 MHz (^1^H frequency) NMR spectrometer equipped with a standard room temperature probe. Similarly, for the residue-specific assignment only four straightforward 3D NMR experiments were sufficient (HNCACB, CBCA(CO)NH, HNCO, HN(CA)CO), and were performed using a single preparation of the sample, which ensured data consistency.

Looking ahead, our methodology offers various options for targeted, segment-specific labeling and efficient investigation of IDR samples. In particular, we envision that SML could be combined with existing ligation techniques such as NCL/EPL/PTS or other emerging enzymatic ligation approaches [65, 66]. This in turn raises the possibility of exploiting orthogonal ligations for inserting segments decorated with various labels within internal regions of large IDR systems for spectroscopic and functional studies.

## Supporting information

S1 Fig Characterization of G_5_-HP60 by analytical ultracentrifugation. (**A**) Results from sedimentation velocity analytical ultracentrifugation showing the c(S) distribution for G_5_-HP60, indicating that G_5_-HP60 is a single species with a sedimentation coefficient of ~1 S. (**B**) Representative result from sedimentation equilibrium analysis of G_5_-HP60 using a single, non-ideal species model with a molecular weight of 7491 Da. Top shows that data as points and the fit as a blue line. Bottom shows the residual plot between the fit and the data points.
(PDF)

S2 Fig Sequences of protein constructs used in this study. (PDF)

S3 Fig SDS-PAGE characterization of unlabeled and uniformly labeled (^13^C/^15^N) proteins. All gels were visualized by Coomassie staining, and molecular weight standards (kDa) are indicated to the left of each gel image. The original, uncropped gel images are also provided in **S14 Fig**.
(PDF)

S4 Fig Sequence and schematic representation of full-length *A*. *thaliana* villin 4. (**A**) Sequence of *A*. *thaliana* villin 4. Positions underlined: V6 domain (621–720) as predicted by homology to V6 domain of chicken villin and HP63 domain (912–974) as determined previously [54, 67]. Positions 721–911 between V6 and HP63 domains represent the disordered linker. (**B**) Schematic representation of villin 4 domains and linker. The N-terminal core fragment (domains V1 through V6, positions 1–720) and C-terminal headpiece domain (HP63) are represented as boxes. The linker (positions 721–911) is shown as a line. The linker has N-terminal basic (positions 721–865, pI 11.5, shown in blue) and C-terminal acidic (877–911, pI 4.1, shown in orange) regions. A predicted PEST motif (866–876, black italics in panel **A**) separates the basic and acidic regions of the linker [55, 56]. For the purposes of the experiments in this study, the C-terminal portion of the villin 4 sequence (residues 877–974) was used.
(PDF)

S5 Fig ESI-MS spectra of model FH8-IDR/GGG ligations. Representative deconvoluted ESI-MS spectra for model sortase-mediated ligation reactions between triglycine (GGG) and (**A**) FH8-IDR (calcd MW unmodified FH8-IDR substrate = 14005 Da, calcd MW ligation product = 13239 Da), (**B**) FH8-IDR-G (calcd MW unmodified FH8-IDR-G substrate = 14062 Da, calcd MW ligation product = 13296 Da), (**C**) FH8-IDR-G_3_ (calcd MW unmodified FH8-IDR-G_3_ substrate = 14176 Da, calcd MW ligation product = 13411 Da), or (**D**) FH8-IDR-(G_4_S)_2_ (calcd MW unmodified FH8-IDR-(G_4_S)_2_ substrate = 14636 Da, calcd MW ligation product = 13870 Da). All spectra represent the 240 min reaction time point. Calculated MW values are average molecular weight predicted using the BMRB Molecular Mass Calculator. * = MeCN adducts from LC-ESI-MS mobile phase (calcd Δ_mass_ for MeCN adduct = +41 Da).
(PDF)

S6 Fig Comparison of SML efficiency for model FH8/GGG ligations. (**A**) C-terminal residues present in FH8-EDEED and FH8-IDR-(G_4_S)_2_. In FH8-EDEED, acidic residues are directly N-terminal to the sortase substrate motif (LPETG). In FH8-IDR-(G_4_S)_2_, acidic residues are separated from the LPETG motif by a neutral, flexible linker. (**B**) Time course of reaction progress for model sortase-mediated ligation reactions between triglycine (GGG) and FH8-IDR-(G_4_S)_2_ or FH8-EDEED [Conditions: 50 μM FH8 substrate, 10 mM triglycine (GGG), 10 μM SrtA_7M_, PIPES buffer (20 mM PIPES, 50 mM NaCl, pH 6.8), 4 h at room temperature]. Reaction progress was estimated using LC-ESI-MS. (**C**) Representative ESI-MS spectrum for ligation reaction between triglycine (GGG) and FH8-EDEED (calcd MW unmodified FH8-EDEED substrate = 11257 Da, calcd MW ligation product = 10491 Da). (**D**) Representative ESI-MS spectrum for ligation reaction between triglycine (GGG) and FH8-IDR-(G_4_S)_2_ (calcd MW unmodified FH8-IDR-(G_4_S)_2_ substrate = 14636 Da, calcd MW ligation product = 13870 Da). ESI-MS spectra in **B** and **C** represent the 30 min reaction time point. Calculated MW values are average molecular weight predicted using the BMRB Molecular Mass Calculator. * = MeCN adducts from LC-ESI-MS mobile phase (calcd Δ_mass_ for MeCN adduct = +41 Da).
(PDF)

S7 Fig ESI-MS and SDS-PAGE characterization of segmentally labeled FH8-IDR-HP63 and IDR-HP63. (**A**) Characterization data for FH8-IDR-HP63 with selective incorporation of ^15^N in the HP63 segment (calcd MW assuming 100% ^15^N incorporation in HP63 = 20797 Da). (**B**) Characterization data for FH8-IDR-HP63 with selective incorporation of ^15^N in the FH8-IDR segment (calcd MW assuming 100% ^15^N incorporation in FH8-IDR = 20859 Da). (**C**) Characterization data for IDR-HP63 (FH8 tag removed) with selective incorporation of ^15^N in the IDR segment (calcd MW assuming 100% ^15^N incorporation in IDR = 12202 Da). Calculated MW values are average molecular weight predicted using the BMRB Molecular Mass Calculator. * = MeCN adducts from LC-ESI-MS mobile phase (calcd Δ_mass_ for MeCN adduct = +41 Da). All gels were visualized by Coomassie staining, and molecular weight standards (kDa) are indicated to the left of each gel image. The original, uncropped gel images are also provided in **S14 Fig**.
(PDF)

S8 Fig Comparison of ^15^N-HSQC spectra for isolated versus segmentally labeled headpiece. **(A**) Overlaid ^15^N-HSQC spectra for isolated, uniformly ^15^N-labeled GG-HP63 (blue contours) and FH8-IDR-HP63 segmentally labeled with ^15^N in the HP63 domain (red contours). The two spectra are nearly identical, with the majority of resonances overlapping or being directly adjacent. Only four pairs of signals deviate from this pattern (indicated with arrowed brackets). The largest observed difference (*) corresponds to leucine residue L899 (numbering based on wild type villin 4), which directly follows the N-terminal, non-native diglycine of GG-HP63. Thus, in isolated GG-HP63 L899 can experience significant end effects due to its location just three positions from the free N-terminus. In segmentally labeled FH8-IDR-HP63, L899 is an internal residue far removed from either terminus. (**B**) atVHP76 (uniform ^15^N labeling, villin 4 headpiece residues 899–974, Biological Magnetic Resonance Data Bank entry 30289) [54].
(PDF)

S9 Fig ^15^N-HSQC of IDR-HP(877–974) (uniform ^15^N labeling). Data recorded at 25°C on a 500 MHz instrument. This sample represents the native sequence of *A*. *thaliana* villin 4 (residues 877–974), and includes the 35-residue IDR segment and C-terminal headpiece domain without the sortase-mediated ligation motif (LPXTG), FH8 domain, or the TEV cleavage site. The only non-native element of IDR-HP(877–974) is the N-terminal His_6_ tag (see **S2** and **S11 Figs**). Based on the sequence of IDR-HP(877–974), we expect to observe up to 101 resonances originating from non-proline residues. A conservative analysis of the spectrum acquired gives 88 resonances of variable intensity. We attribute this apparent shortage in part to the presence of the His_6_ tag on the protein N-terminus. Due to their identical chemical nature, these six contiguous residues would likely have nearly overlapping or closely packed peaks. Additionally, there is a region of poor spectral resolution at ~8.3 ppm (^1^H dimension) and ~122 ppm (^15^N dimension) where significant spectral overlap may prevent identification of more individual peaks. Lastly, resonances may be undetectable due to their respective residues experiencing intermediate time-scale dynamics.
(PDF)

S10 Fig ^15^N-HSQC spectra of isolated FH8 (uniform ^15^N labeling). **(A)** ^15^N-HSQC spectra of the isolated FH8 domain (at 500 MHz ^1^H frequency) recorded at 25°C. For comparison, the ^15^N-HSQC spectrum of FH8-IDR-HP63 at 25°C (segmentally labeled with ^15^N in the FH8-IDR segment) is shown in panel **B**.
(PDF)

S11 Fig Overlaid ^15^N-HSQC spectra of FH8-IDR-HP63 and IDR-HP(877–974). Superimposed ^15^N-HSQC spectra for FH8-IDR-HP63 segmentally labeled with ^13^C/^15^N in the FH8-IDR portion (red, recorded on a 600 MHz ^1^H frequency instrument) and uniformly ^15^N-labeled IDR-HP(877–974) (native sequence control, open blue contours, same spectrum as **S9 Fig**, recorded at 500 MHz ^1^H frequency). The filled red contours indicate segmentally assigned FH8-IDR-HP63 resonances (see **Fig 5** in main text). An “X” signifies the eight FH8-IDR-HP63 resonances for which there are no overlapping or adjacent resonances in the IDR-HP(877–974) control. Seven out of these eight FH8-IDR-HP63 resonances were assigned to the introduced TEV site (L_74_Y_75_F_76_Q_77_G_78_), the single glycine spacer (G_114_), or the leucine (L_115_) of the sortase ligation site. Double arrows indicate four segmentally assigned resonances (solid red ovals) for which there are nearby matching signals in the native control (open blue contours). The length of every arrow is equal or smaller than 0.05 ppm in the ^1^H dimension. Primary structure diagrams are shown below the spectra for segmentally labeled FH8-IDR-H63 (sequence position numbering begins with the first residue of the FH8 domain) and uniformly labeled IDR-HP(877–974) (residue numbering corresponds to the sequence of native villin 4). Non-black coloration in the structure diagrams (red or blue) indicates the position of isotopic labels (^13^C and/or ^15^N).
(PDF)

S12 Fig ^15^N-HSQC spectra of G_5_-HP60. ^15^N-HSQC spectra of isolated G_5_-HP60 (solid red contours, uniform ^15^N labeling) at (**A**) 25°C and (**B**) 5°C. For comparison, each spectrum of G_5_-HP60 is overlaid with the ^15^N-HSQC spectrum of atVHP60 (25°C, open blue contours, atVHP60 spectrum reproduced from Miears et al.) [54]. The spectra were recorded at 500 MHz (^1^H frequency). Circled resonances correspond to amino acid side chains. The sequences of G_5_-HP60 and atVHP60 are shown at the bottom (coloration matches the corresponding signals in the overlaid spectra). Underlined residues correspond to non-native amino acids added to the HP60 domain. In the case of G_5_-HP60, five glycines were included to ensure accessibility of the N-terminus for potential sortase-mediated ligation.
(PDF)

S13 Fig ^15^N-HSQC NMR spectra (500 MHz, ^1^H frequency) of segmentally labeled IDR-HP63 (^15^N-labeled IDR segment). Spectra recorded at (**A**) 15°C, (**B**) 25°C, and (**C**) 45°C. The spectra recorded at 15°C and 45°C were re-referenced to the ^1^H / H_2_O chemical shift value of the spectrum recorded at 25°C according to the standard approach [63]. Residue specific assignments in panels **A** and **C** were transferred from the spectrum acquired at 25°C (see **Fig 5B**). Peaks for which no residue specific assignments were obtained are marked with “+” or “x”, with “+” indicating unassigned peaks that were observed in the FH8-IDR-HP63 construct (^13^C/^15^N-labeled FH8-IDR segment), and “x” indicating unassigned peaks unique to the IDR-HP63 derivative (^15^N-labeled IDR segment). Two of the peaks marked “x” are presumed to correspond to resonances for residues E79 and E80 (adjacent to G78 at the N-terminus in the IDR-HP63 sample).
(PDF)

S14 Fig Raw gel images for S3 and S7 Figs. (PDF)

S1 Table ESI-MS Characterization of Unlabeled and Uniformly Labeled (^15^N) Proteins. ^*a*^Calculated values are average molecular weight predicted using the BMRB Molecular Mass Calculator [68]. For isotopically labeled samples, calculated masses assume 100% incorporation of ^15^N. ^*b*^A secondary mass peak of lower intensity was observed at 18158 Da, which is consistent with the presence of a glutathione adduct (calcd mass for glutathione adduct = 18157 Da) [69]. No evidence was found that this adduct impeded SML reactions, and this enzyme preparation was used without additional purification. ^*c*^Mass of FH8-IDR-G doubly labeled with ^13^C and ^15^N was not determined.
(PDF)

S2 Table Summary of ^15^N-HSQC spectral changes attributed to the presence of the FH8 tag and non-native residues (GLPETGG) inserted for sortase-mediated ligation. ^*a*^Residue number based on position in the FH8-IDR-HP63 construct. Value in parentheses indicates the corresponding position in wild-type villin 4. The native 35-residue portion of the IDR covers position E79 (E877)–D113 (D911). ^*b*^Effect of FH8 assessed by comparison of ^15^N-HSQC spectra for FH8-IDR-HP63 (^13^C/^15^N-labeling restricted to FH8-IDR segment, as in **Fig 5A**) and IDR-HP63 (^15^N-labeling restricted to IDR segment, as in **Fig 5B**). All spectra recorded at 25°C. ^*c*^Effect of GLPETGG insertion assessed by comparison of spectra for FH8-IDR-HP63 and IDR-HP(877–974) (uniformly ^15^N-labeled, as in **S11 Fig**). All spectra recorded at 25°C. ^*d*^Observed chemical shift differences attributed to the presence of the His_6_-tag on the N-terminus of IDR-HP(877–974) as opposed to the non-native, N-terminal G78 residue in the case of IDR-HP63. ^*e*^*Nearly no effect* indicates chemical shift differences of <0.04 ppm for the ^1^H dimension and <0.2 ppm for the ^15^N dimension. ^*f*^Alignment of relevant sequences (35-residue IDR fragment indicated in **bold**).
(PDF)

## Abbreviations

SML: sortase-mediated ligation
IDR: intrinsically disordered region
NMR: nuclear magnetic resonance
HSQC: spectra, heteronuclear single quantum coherence spectra
LC-ESI-MS: liquid chromatography electrospray ionization mass spectrometry
