# Supplementary figures and images for: Sortase-mediated segmental labeling: A method for segmental assignment of intrinsically disordered regions in proteins
Source: PLoS One. 2021 Oct 28;16(10):e0258531. doi: 10.1371/journal.pone.0258531 (PMC8553144; doi:10.1371/journal.pone.0258531)

**S1 Figure**

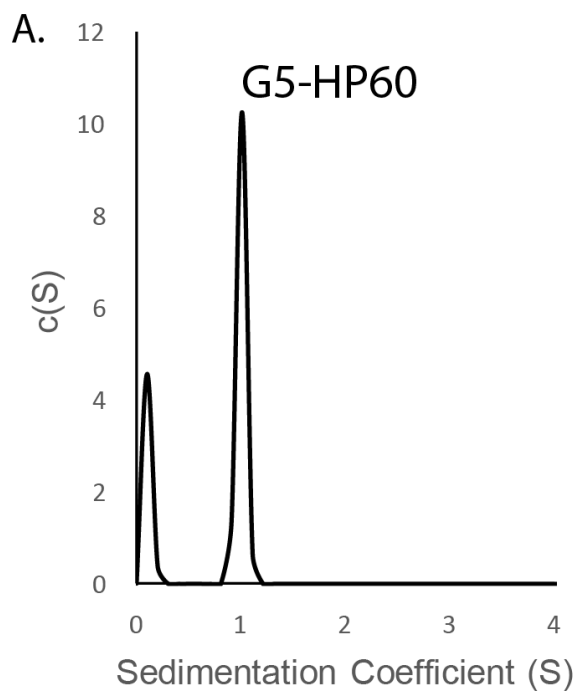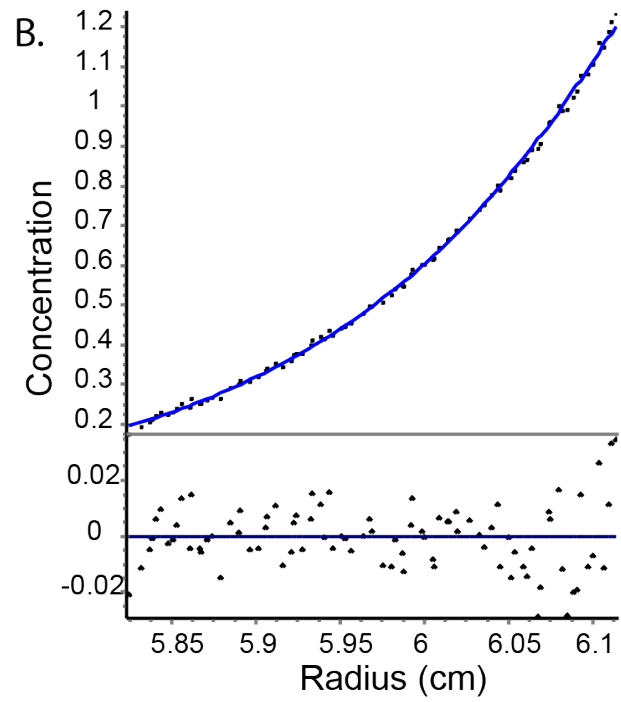

Supplement: S1 Fig — (A) Results from sedimentation velocity analytical ultracentrifugation showing the c(S) distribution for G5-HP60, indicating that G5-HP60 is a single species with a sedimentation coefficient of ~1 S. (B) Representative result from sedimentation equilibrium analysis of G5-HP60 using a single, non-ideal species model with a molecular weight of 7491 Da. Top shows that data as points and the fit as a blue line. Bottom shows the residual plot between the fit and the data points. (PDF) [file pone.0258531.s001.pdf]

S3 Figure

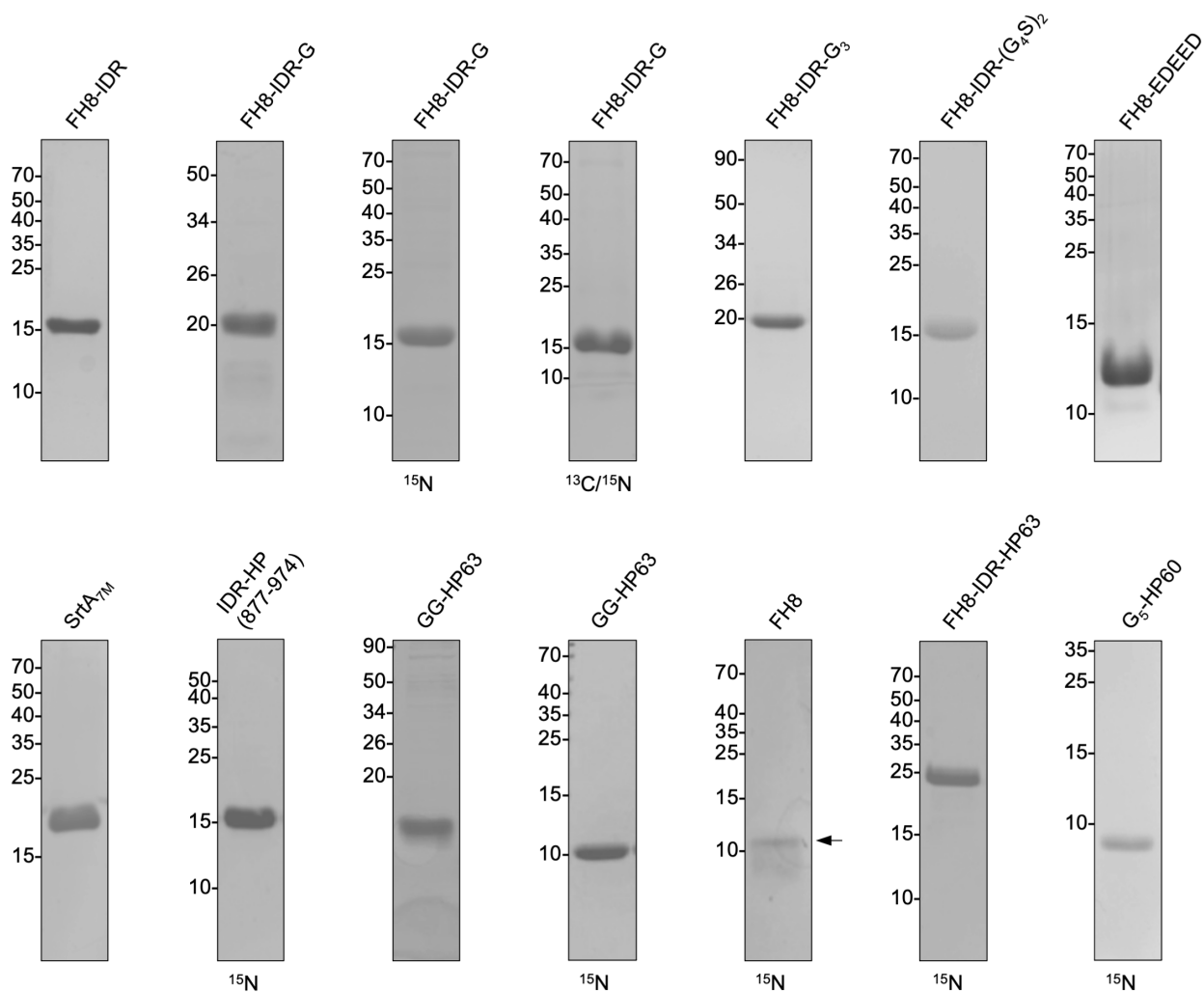

Supplement: S3 Fig — All gels were visualized by Coomassie staining, and molecular weight standards (kDa) are indicated to the left of each gel image. The original, uncropped gel images are also provided in S14 Fig. (PDF) [file pone.0258531.s003.pdf]

## S5 Figure

**A** FH8-IDR + GGG

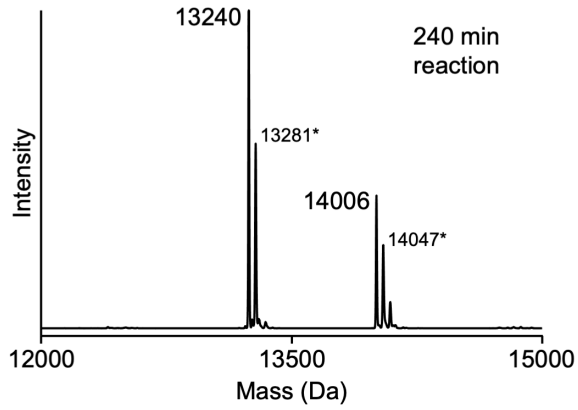

**B** FH8-IDR-G + GGG

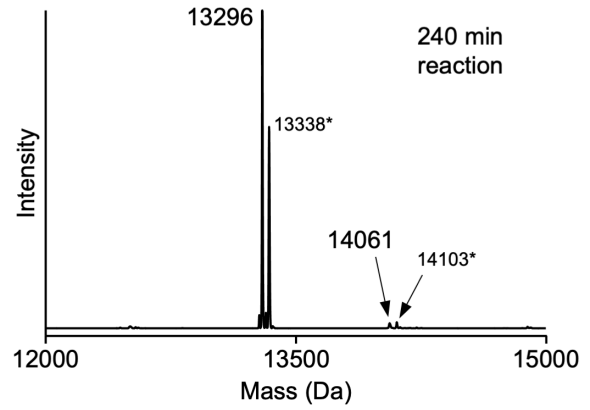

**C** FH8-IDR-G<sub>3</sub> + GGG

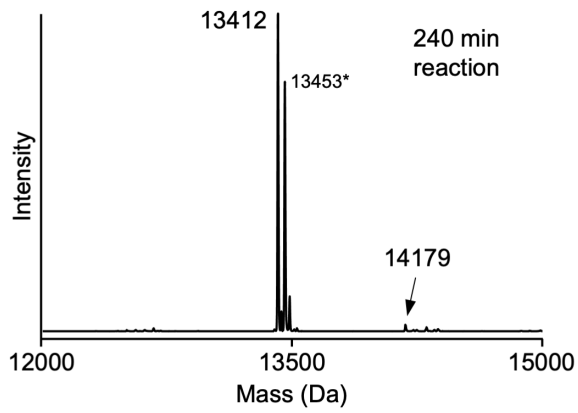

**D** FH8-IDR-(G<sub>4</sub>S)<sub>2</sub> + GGG

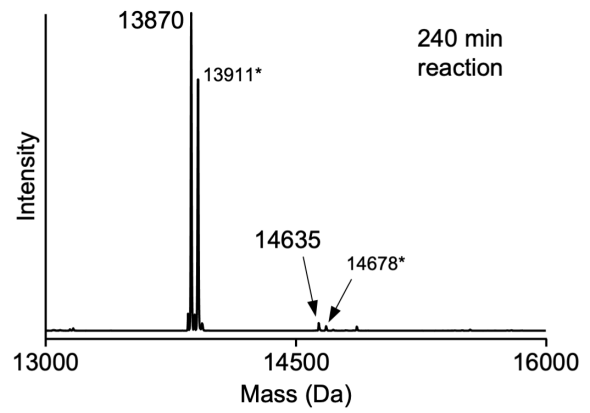

Supplement: S5 Fig — Representative deconvoluted ESI-MS spectra for model sortase-mediated ligation reactions between triglycine (GGG) and (A) FH8-IDR (calcd MW unmodified FH8-IDR substrate = 14005 Da, calcd MW ligation product = 13239 Da), (B) FH8-IDR-G (calcd MW unmodified FH8-IDR-G substrate = 14062 Da, calcd MW ligation product = 13296 Da), (C) FH8-IDR-G3 (calcd MW unmodified FH8-IDR-G3 substrate = 14176 Da, calcd MW ligation product = 13411 Da), or (D) FH8-IDR-(G4S)2 (calcd MW unmodified FH8-IDR-(G4S)2 substrate = 14636 Da, calcd MW ligation product = 13870 Da). All spectra represent the 240 min reaction time point. Calculated MW values are average molecular weight predicted using the BMRB Molecular Mass Calculator. * = MeCN adducts from LC-ESI-MS mobile phase (calcd Δmass for MeCN adduct = +41 Da). (PDF) [file pone.0258531.s005.pdf]

## S6 Figure

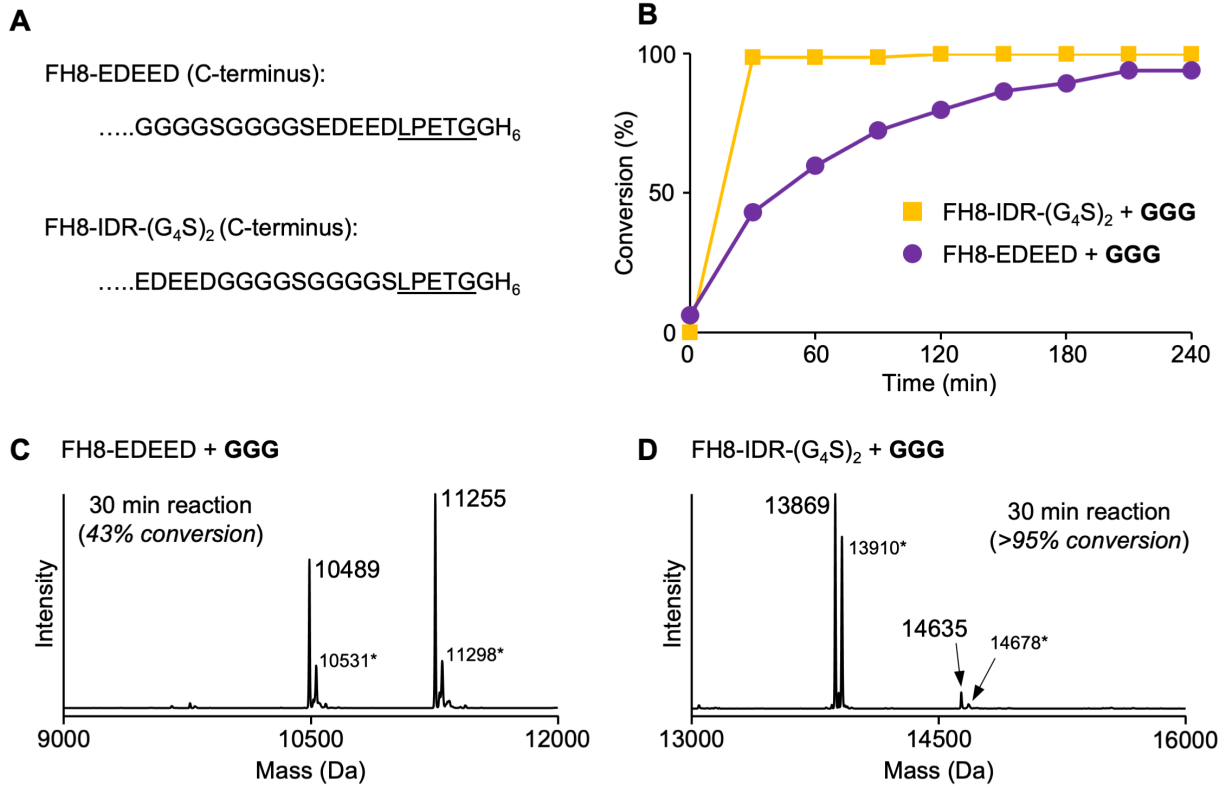

Supplement: S6 Fig — (A) C-terminal residues present in FH8-EDEED and FH8-IDR-(G4S)2. In FH8-EDEED, acidic residues are directly N-terminal to the sortase substrate motif (LPETG). In FH8-IDR-(G4S)2, acidic residues are separated from the LPETG motif by a neutral, flexible linker. (B) Time course of reaction progress for model sortase-mediated ligation reactions between triglycine (GGG) and FH8-IDR-(G4S)2 or FH8-EDEED [Conditions: 50 μM FH8 substrate, 10 mM triglycine (GGG), 10 μM SrtA7M, PIPES buffer (20 mM PIPES, 50 mM NaCl, pH 6.8), 4 h at room temperature]. Reaction progress was estimated using LC-ESI-MS. (C) Representative ESI-MS spectrum for ligation reaction between triglycine (GGG) and FH8-EDEED (calcd MW unmodified FH8-EDEED substrate = 11257 Da, calcd MW ligation product = 10491 Da). (D) Representative ESI-MS spectrum for ligation reaction between triglycine (GGG) and FH8-IDR-(G4S)2 (calcd MW unmodified FH8-IDR-(G4S)2 substrate = 14636 Da, calcd MW ligation product = 13870 Da). ESI-MS spectra in B and C represent the 30 min reaction time point. Calculated MW values are average molecular weight predicted using the BMRB Molecular Mass Calculator. * = MeCN adducts from LC-ESI-MS mobile phase (calcd Δmass for MeCN adduct = +41 Da). (PDF) [file pone.0258531.s006.pdf]

S7 Figure

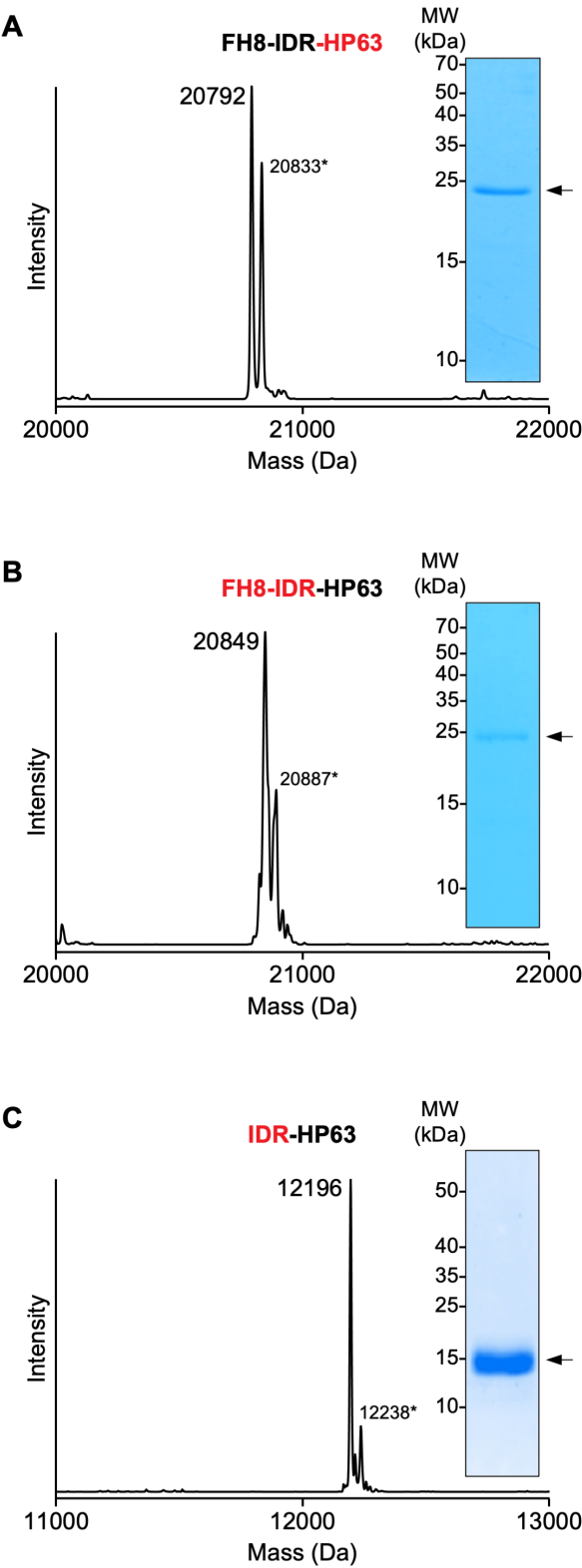

Supplement: S7 Fig — (A) Characterization data for FH8-IDR-HP63 with selective incorporation of 15N in the HP63 segment (calcd MW assuming 100% 15N incorporation in HP63 = 20797 Da). (B) Characterization data for FH8-IDR-HP63 with selective incorporation of 15N in the FH8-IDR segment (calcd MW assuming 100% 15N incorporation in FH8-IDR = 20859 Da). (C) Characterization data for IDR-HP63 (FH8 tag removed) with selective incorporation of 15N in the IDR segment (calcd MW assuming 100% 15N incorporation in IDR = 12202 Da). Calculated MW values are average molecular weight predicted using the BMRB Molecular Mass Calculator. * = MeCN adducts from LC-ESI-MS mobile phase (calcd Δmass for MeCN adduct = +41 Da). All gels were visualized by Coomassie staining, and molecular weight standards (kDa) are indicated to the left of each gel image. The original, uncropped gel images are also provided in S14 Fig. (PDF) [file pone.0258531.s007.pdf]

**S8 Figure**

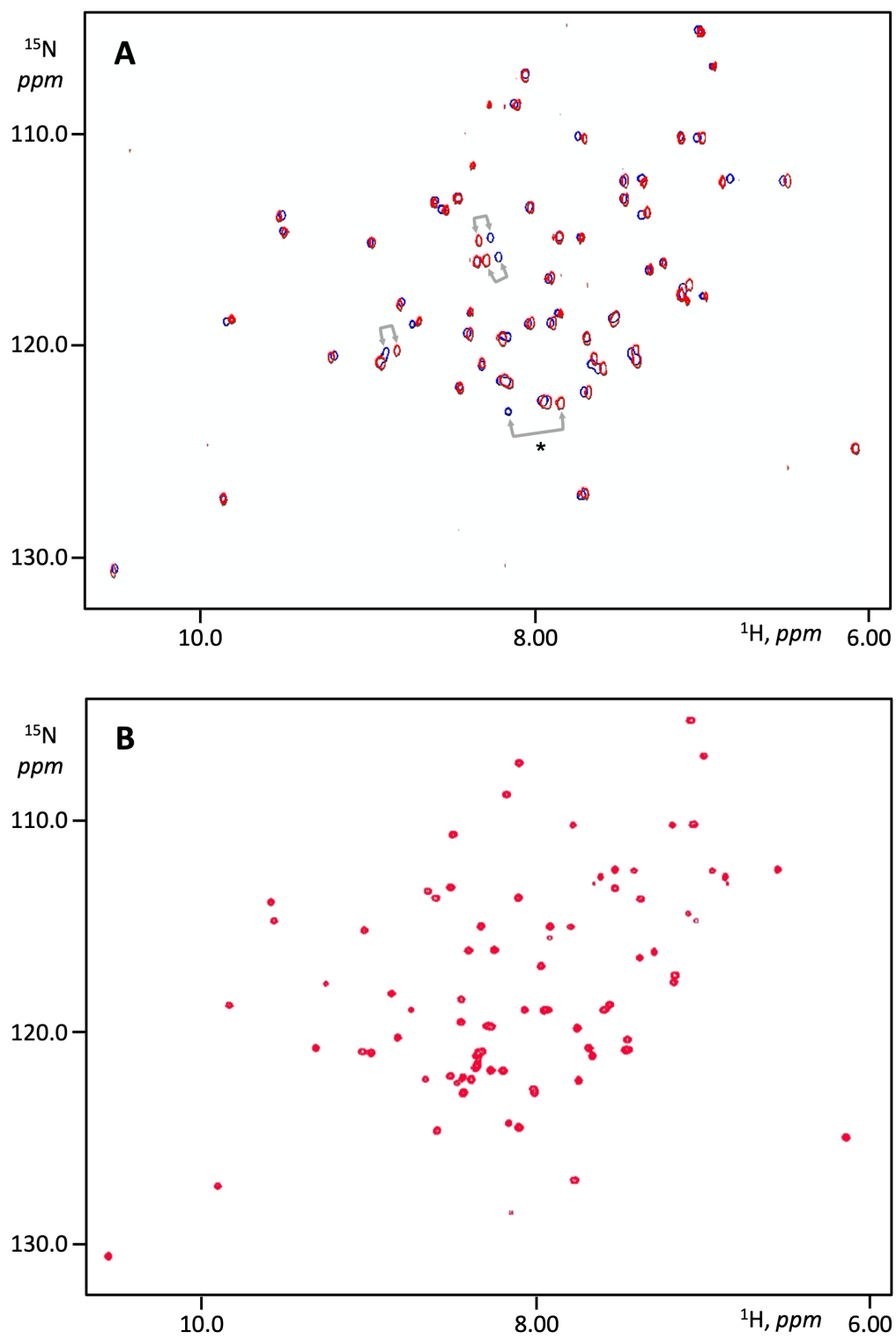

Supplement: S8 Fig — (A) Overlaid 15N-HSQC spectra for isolated, uniformly 15N-labeled GG-HP63 (blue contours) and FH8-IDR-HP63 segmentally labeled with 15N in the HP63 domain (red contours). The two spectra are nearly identical, with the majority of resonances overlapping or being directly adjacent. Only four pairs of signals deviate from this pattern (indicated with arrowed brackets). The largest observed difference (*) corresponds to leucine residue L899 (numbering based on wild type villin 4), which directly follows the N-terminal, non-native diglycine of GG-HP63. Thus, in isolated GG-HP63 L899 can experience significant end effects due to its location just three positions from the free N-terminus. In segmentally labeled FH8-IDR-HP63, L899 is an internal residue far removed from either terminus. (B) atVHP76 (uniform 15N labeling, villin 4 headpiece residues 899–974, Biological Magnetic Resonance Data Bank entry 30289) [54]. (PDF) [file pone.0258531.s008.pdf]

**S9 Figure**

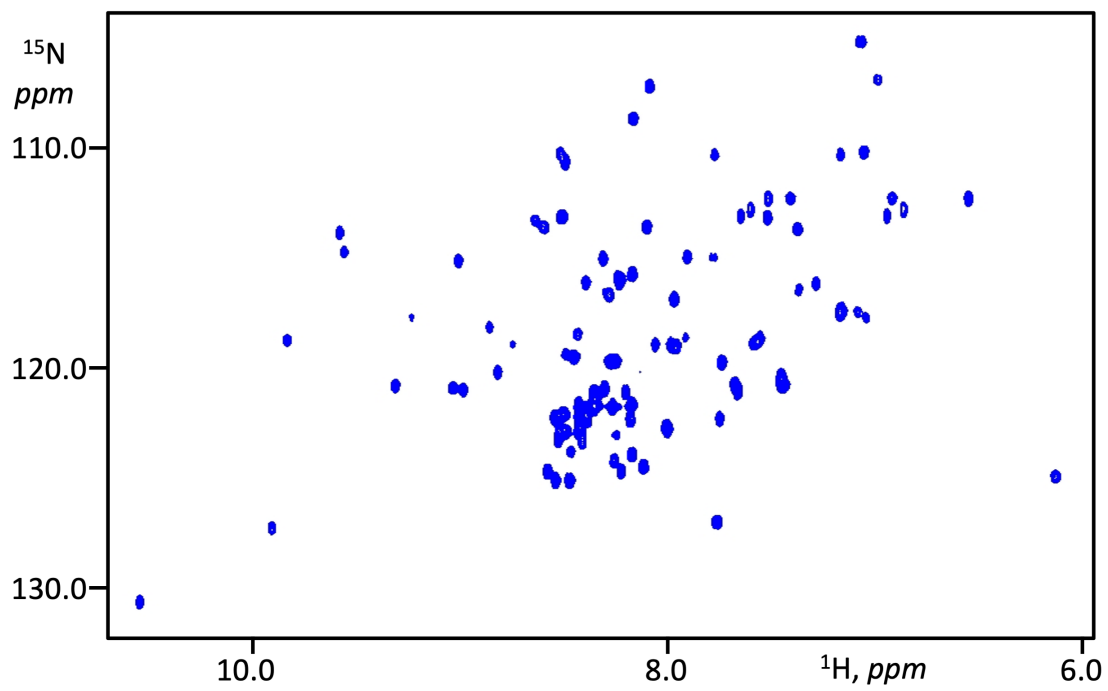

Supplement: S9 Fig — Data recorded at 25°C on a 500 MHz instrument. This sample represents the native sequence of A. thaliana villin 4 (residues 877–974), and includes the 35-residue IDR segment and C-terminal headpiece domain without the sortase-mediated ligation motif (LPXTG), FH8 domain, or the TEV cleavage site. The only non-native element of IDR-HP(877–974) is the N-terminal His6 tag (see S2 and S11 Figs). Based on the sequence of IDR-HP(877–974), we expect to observe up to 101 resonances originating from non-proline residues. A conservative analysis of the spectrum acquired gives 88 resonances of variable intensity. We attribute this apparent shortage in part to the presence of the His6 tag on the protein N-terminus. Due to their identical chemical nature, these six contiguous residues would likely have nearly overlapping or closely packed peaks. Additionally, there is a region of poor spectral resolution at ~8.3 ppm (1H dimension) and ~122 ppm (15N dimension) where significant spectral overlap may prevent identification of more individual peaks. Lastly, resonances may be undetectable due to their respective residues experiencing intermediate time-scale dynamics. (PDF) [file pone.0258531.s009.pdf]

**S10 Figure**

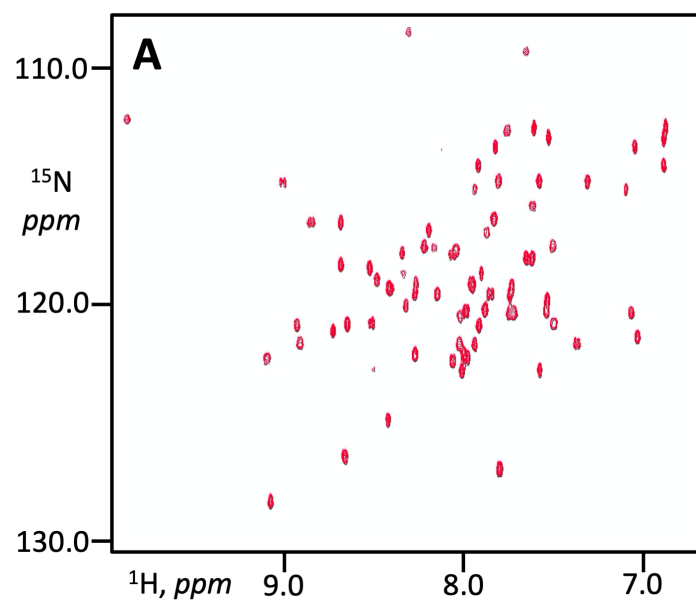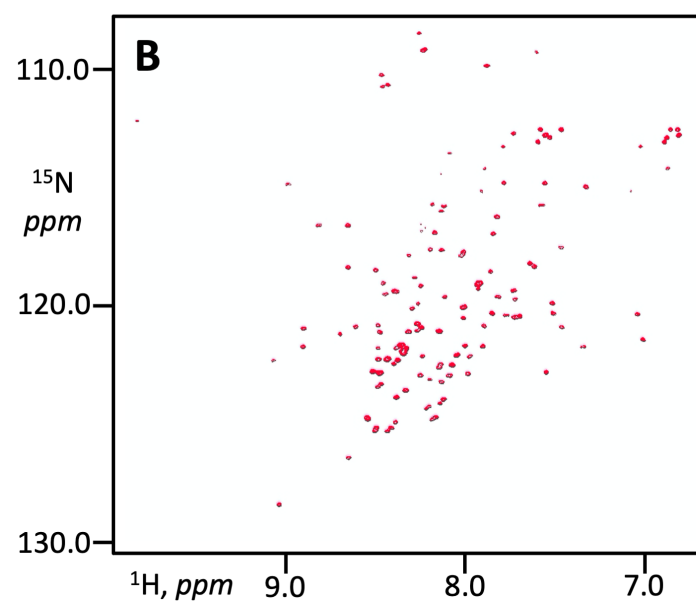

Supplement: S10 Fig — (A) 15N-HSQC spectra of the isolated FH8 domain (at 500 MHz 1H frequency) recorded at 25°C. For comparison, the 15N-HSQC spectrum of FH8-IDR-HP63 at 25°C (segmentally labeled with 15N in the FH8-IDR segment) is shown in panel B. (PDF) [file pone.0258531.s010.pdf]

S11 Figure

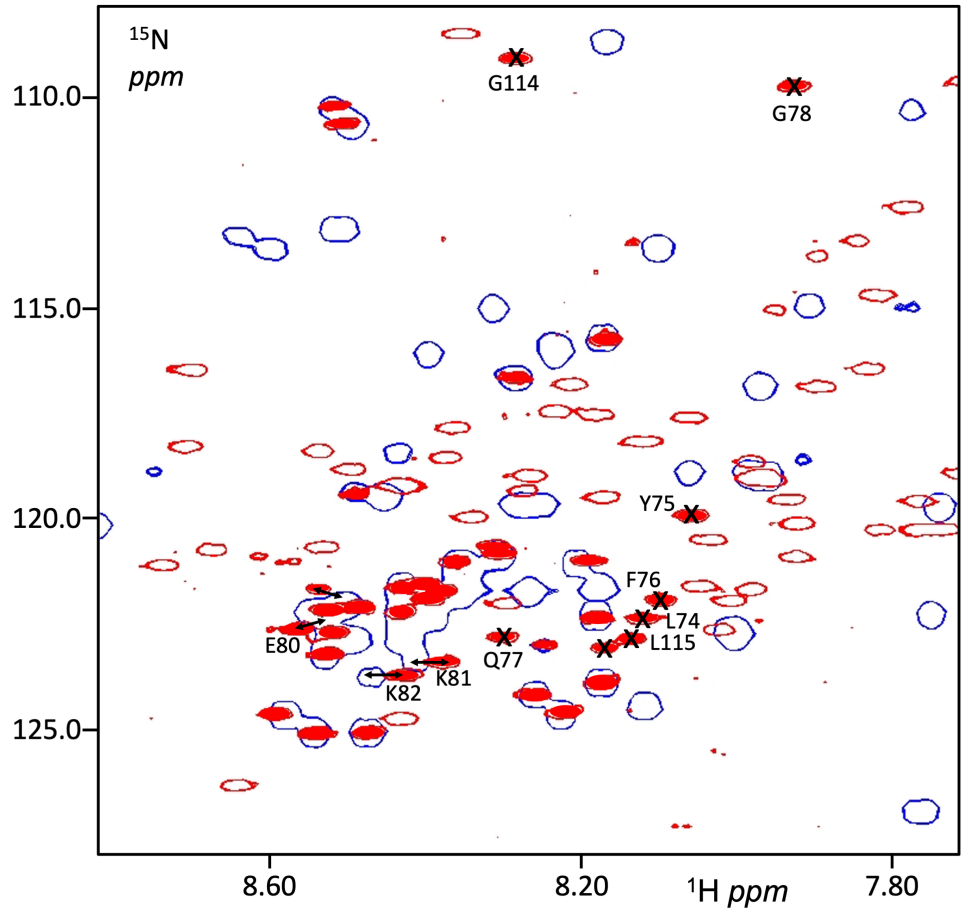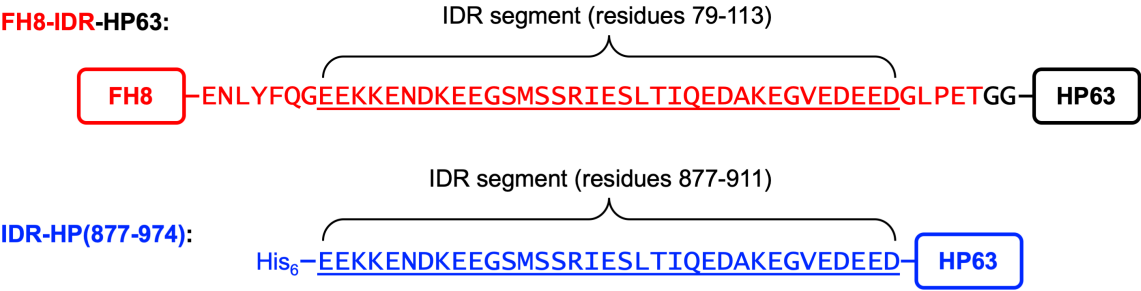

Supplement: S11 Fig — Superimposed 15N-HSQC spectra for FH8-IDR-HP63 segmentally labeled with 13C/15N in the FH8-IDR portion (red, recorded on a 600 MHz 1H frequency instrument) and uniformly 15N-labeled IDR-HP(877–974) (native sequence control, open blue contours, same spectrum as S9 Fig, recorded at 500 MHz 1H frequency). The filled red contours indicate segmentally assigned FH8-IDR-HP63 resonances (see Fig 5 in main text). An “X” signifies the eight FH8-IDR-HP63 resonances for which there are no overlapping or adjacent resonances in the IDR-HP(877–974) control. Seven out of these eight FH8-IDR-HP63 resonances were assigned to the introduced TEV site (L74Y75F76Q77G78), the single glycine spacer (G114), or the leucine (L115) of the sortase ligation site. Double arrows indicate four segmentally assigned resonances (solid red ovals) for which there are nearby matching signals in the native control (open blue contours). The length of every arrow is equal or smaller than 0.05 ppm in the 1H dimension. Primary structure diagrams are shown below the spectra for segmentally labeled FH8-IDR-H63 (sequence position numbering begins with the first residue of the FH8 domain) and uniformly labeled IDR-HP(877–974) (residue numbering corresponds to the sequence of native villin 4). Non-black coloration in the structure diagrams (red or blue) indicates the position of isotopic labels (13C and/or 15N). (PDF) [file pone.0258531.s011.pdf]

**S13 Figure**

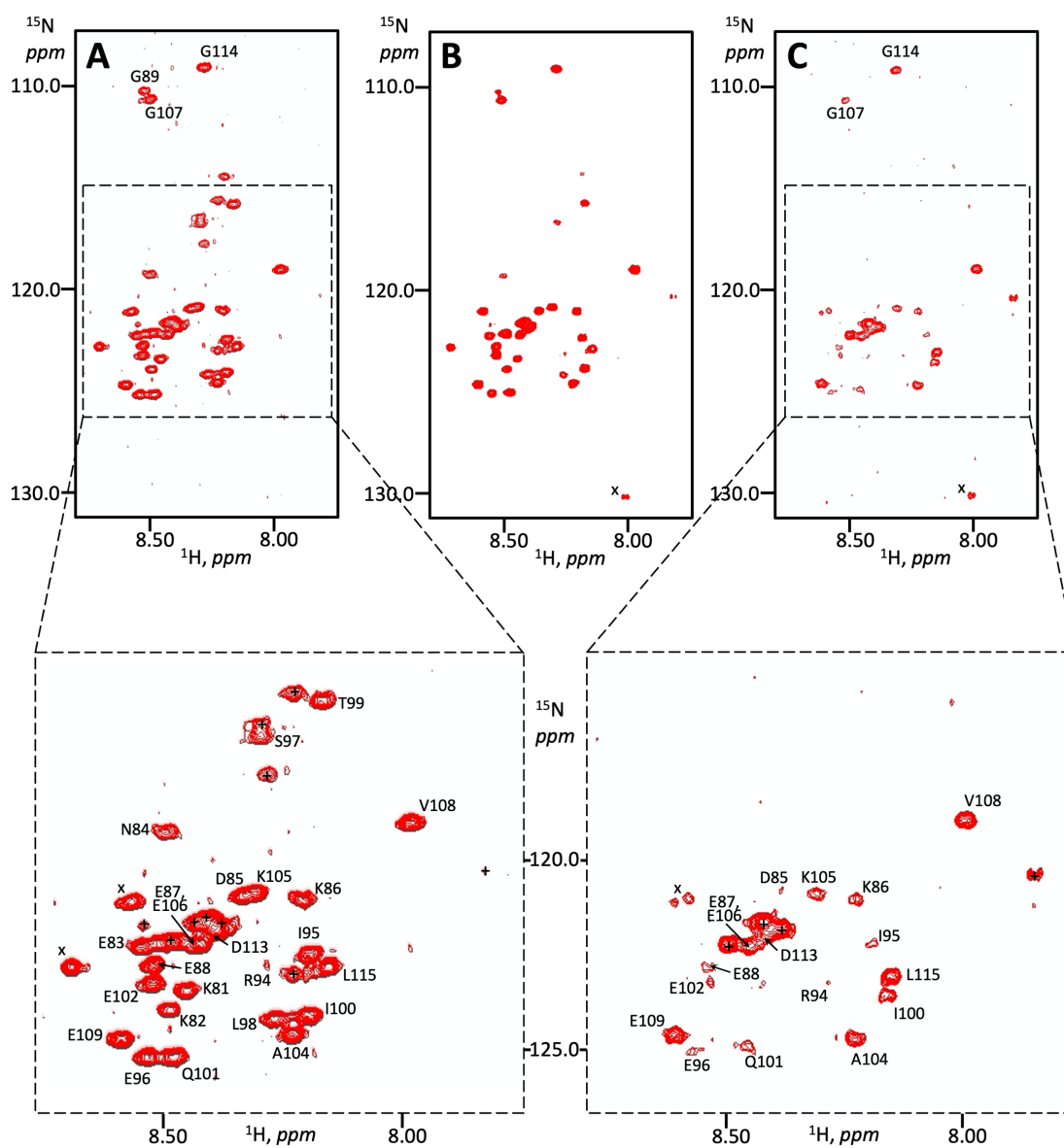

Supplement: S13 Fig — Spectra recorded at (A) 15°C, (B) 25°C, and (C) 45°C. The spectra recorded at 15°C and 45°C were re-referenced to the 1H / H2O chemical shift value of the spectrum recorded at 25°C according to the standard approach [63]. Residue specific assignments in panels A and C were transferred from the spectrum acquired at 25°C (see Fig 5B). Peaks for which no residue specific assignments were obtained are marked with “+” or “x”, with “+” indicating unassigned peaks that were observed in the FH8-IDR-HP63 construct (13C/15N-labeled FH8-IDR segment), and “x” indicating unassigned peaks unique to the IDR-HP63 derivative (15N-labeled IDR segment). Two of the peaks marked “x” are presumed to correspond to resonances for residues E79 and E80 (adjacent to G78 at the N-terminus in the IDR-HP63 sample). (PDF) [file pone.0258531.s013.pdf]

**S14 Figure** (page 1 of 3)

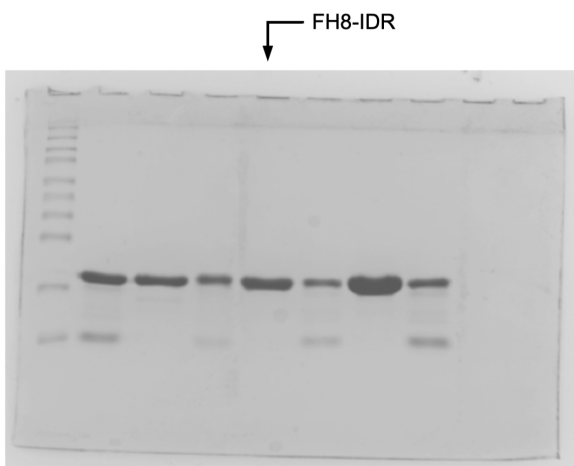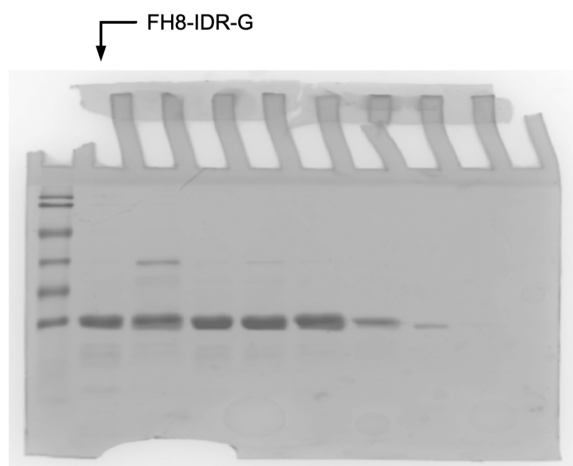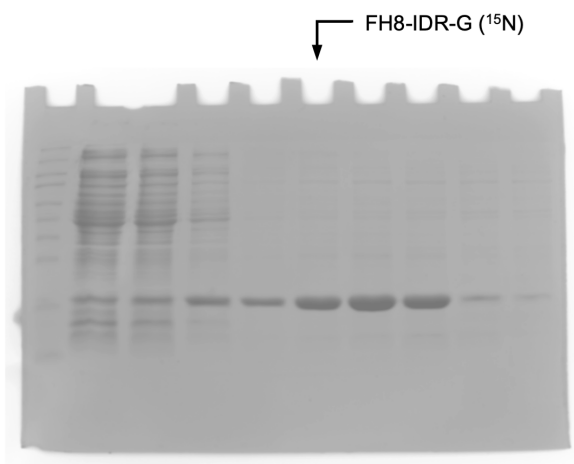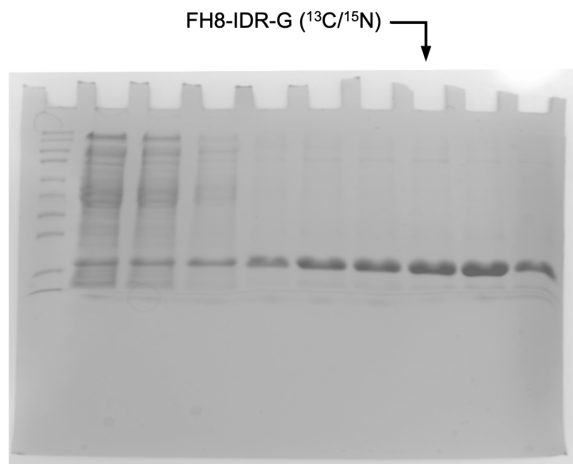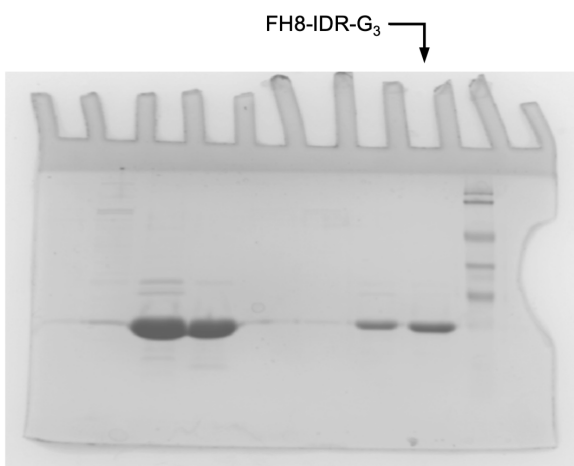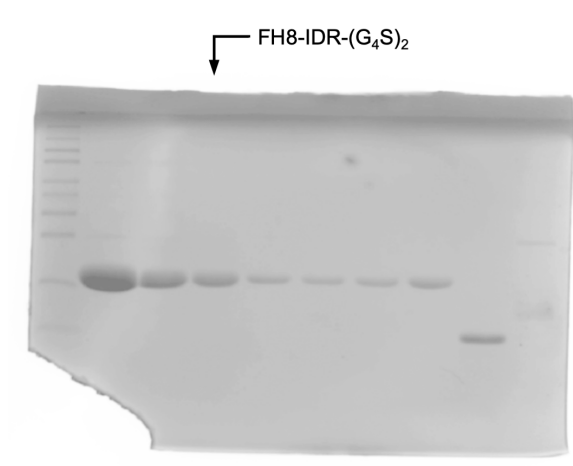

**S14 Figure** (page 2 of 3)

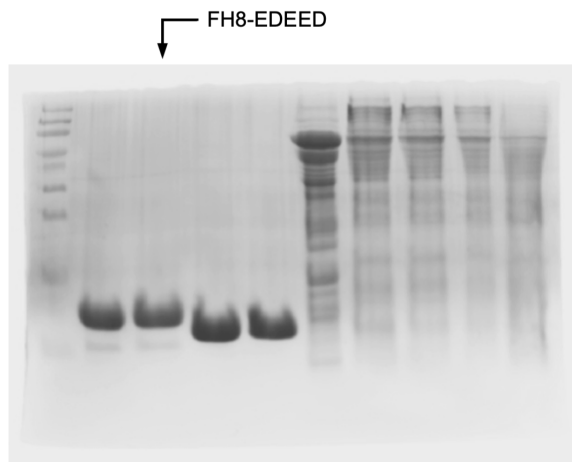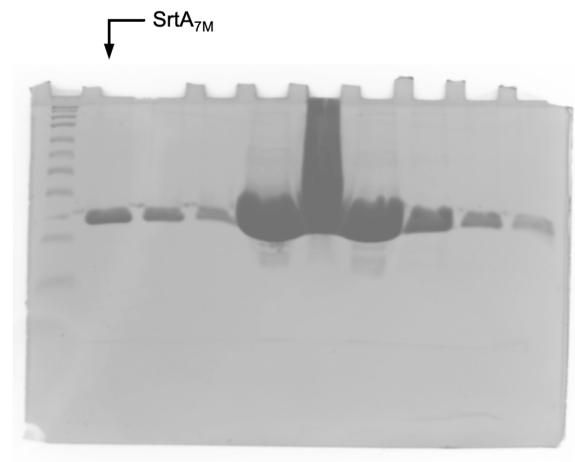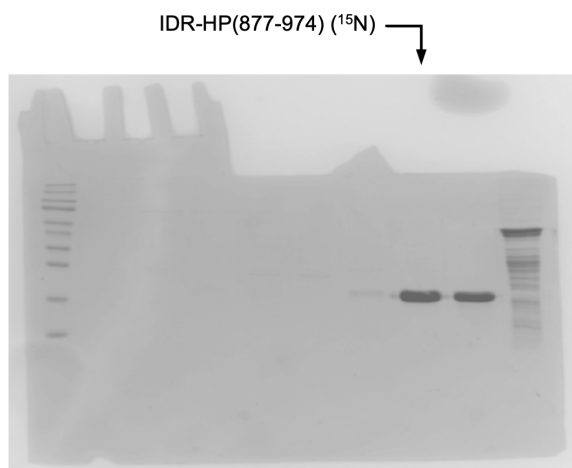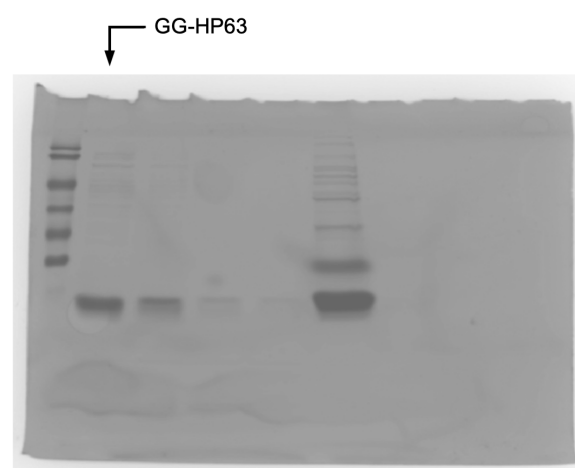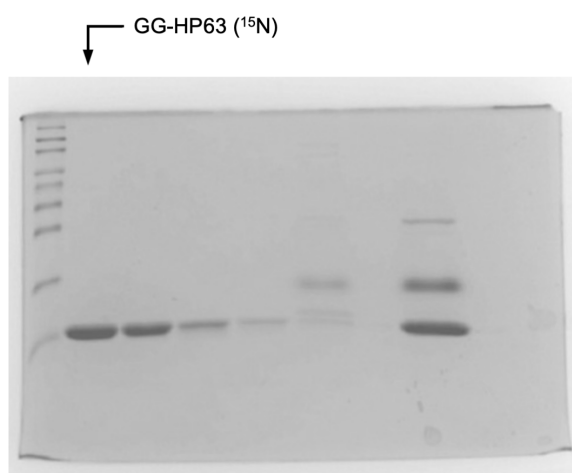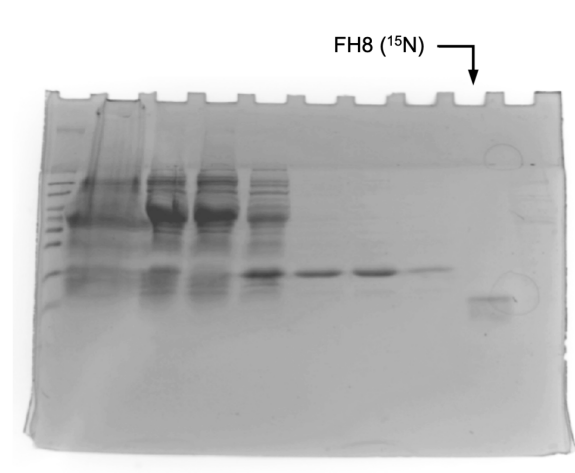

**S14 Figure** (page 3 of 3)

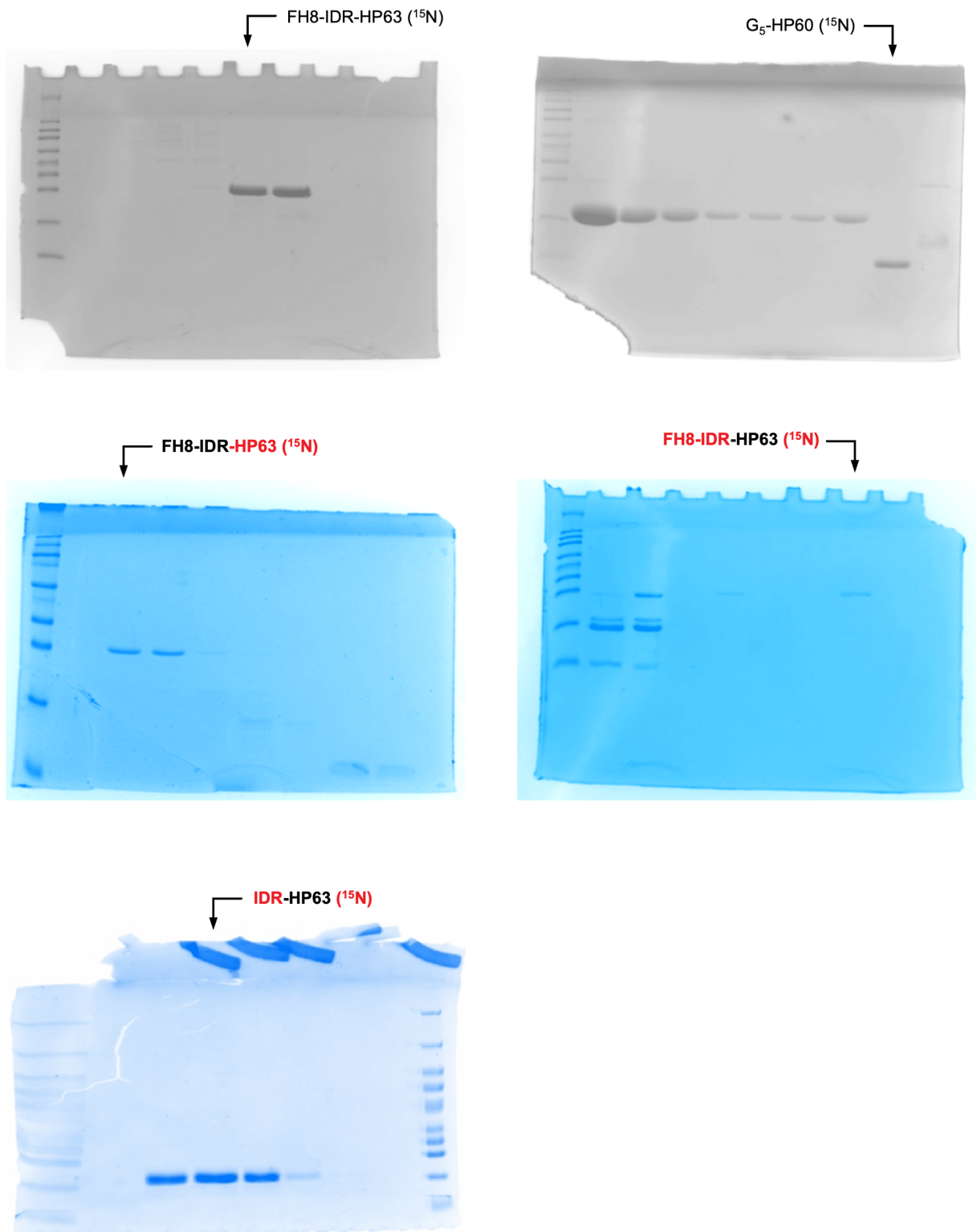

Supplement: S14 Fig — (PDF) [file pone.0258531.s014.pdf]
