# Supplementary material for: Sortase-mediated segmental labeling: A method for segmental assignment of intrinsically disordered regions in proteins
Source: PLoS One. 2021 Oct 28;16(10):e0258531. doi: 10.1371/journal.pone.0258531 (PMC8553144; doi:10.1371/journal.pone.0258531)
Supplement: S2 Fig — (PDF) [file pone.0258531.s002.pdf]

S2 Figure (*LPXTG sortase substrate motifs and TEV cleavage site residues underlined)*

1. **SrtA7M**
MQAKPQIPKDKSKVAGYIEIPDADIKEPVYPGPATREQLNRGVSFAKENQSLDDQNISIAGHTFIDRPNYQF
TNLKAAKKGSMVYFKVGNETRKYKMTSIRNVKPTAVEVLDEQKGKDKQLTLITCDDYNEETGVWETRKIFV
ATEVKLEHHHHHH

2. **TEV-GG-HP63**
MHHHHHHENLYFQGGLPAHPYDRLKTTSTDPVSDIDVTRREAYLSSEEFKEKFGMTKEAFYKLPKWKQNK
FKMAVQLF

3. **GG-HP63** (*generated via TEV protease cleavage of TEV-GG-HP63)*
GGLPAHPYDRLKTTSTDPVSDIDVTRREAYLSSEEFKEKFGMTKEAFYKLPKWKQNKFKMAVQLF

4. **FH8-IDR** *(initiator methionine absent in purified protein)*
PSVQEVEKLLHVLDRNGDGKVSAEELKAFADDSKCPLDSNKIKAFIKEHDKNKDGKLDLKELVSILSSGTSE
NLYFQGEEKKENDKEEGSMSSRIESLTIQEDAKEGVEDEEDLPETGGHHHHHH

5. **FH8-IDR-G** *(initiator methionine absent in purified protein)*
PSVQEVEKLLHVLDRNGDGKVSAEELKAFADDSKCPLDSNKIKAFIKEHDKNKDGKLDLKELVSILSSGTSE
NLYFQGEEKKENDKEEGSMSSRIESLTIQEDAKEGVEDEEDGLPETGGHHHHHH

6. **FH8-IDR-G$_3$** *(initiator methionine absent in purified protein)*
PSVQEVEKLLHVLDRNGDGKVSAEELKAFADDSKCPLDSNKIKAFIKEHDKNKDGKLDLKELVSILSSGTSE
NLYFQGEEKKENDKEEGSMSSRIESLTIQEDAKEGVEDEEDGGGLPETGGHHHHHH

7. **FH8-IDR-(G$_4$S)$_2$** *(initiator methionine absent in purified protein)*
PSVQEVEKLLHVLDRNGDGKVSAEELKAFADDSKCPLDSNKIKAFIKEHDKNKDGKLDLKELVSILSSGTSE
NLYFQGEEKKENDKEEGSMSSRIESLTIQEDAKEGVEDEEDGGGGSGGGGSLPETGGHHHHHH

8. **FH8-EDEED** *(initiator methionine absent in purified protein)*
PSVQEVEKLLHVLDRNGDGKVSAEELKAFADDSKCPLDSNKIKAFIKEHDKNKDGKLDLKELVSILSSGTSE
NLYFQGGGGGSGGGGSEDEEDLPETGGHHHHHH

9. **FH8** *(generated via TEV protease cleavage of FH8-IDR-G)*
PSVQEVEKLLHVLDRNGDGKVSAEELKAFADDSKCPLDSNKIKAFIKEHDKNKDGKLDLKELVSILSSGTSE
NLYFQ

10. **FH8-IDR-HP63** *(full length fusion of villin 4 IDR (residues 877-911) and HP63, generated either through expression of full length protein or via sortase-mediated ligation of FH8-IDR-G and GG-HP63; all contain single G spacer between IDR and LPXTG motif)*
PSVQEVEKLLHVLDRNGDGKVSAEELKAFADDSKCPLDSNKIKAFIKEHDKNKDGKLDLKELVSILSSGTSE
NLYFQGEEKKENDKEEGSMSSRIESLTIQEDAKEGVEDEEDGLPETGGLPAHPYDRLKTTSTDPVSDIDVT
RREAYLSSEEFKEKFGMTKEAFYKLPKWKQNKFKMAVQLF

11. **IDR-HP63** *(generated via TEV protease cleavage of FH8-IDR-HP63)*
GEEKKENDKEEGSMSSRIESLTIQEDAKEGVEDEEDGLPETGGLPAHPYDRLKTTSTDPVSDIDVTRREAY
LSSEEFKEKFGMTKEAFYKLPKWKQNKFKMAVQLF

12. **IDR-HP(877-974)** *(native sequence control sample lacking sortase LPXTG motif, TEV site, and FH8 domain)*
MHHHHHHEEKKENDKEEGSMSSRIESLTIQEDAKEGVEDEEDLPAHPYDRLKTTSTDPVSDIDVTRREAYL
SSEEFKEKFGMTKEAFYKLPKWKQNKFKMAVQLF

13. **IDR-G$_5$-HP60**
MHHHHHHEEKKENDKEEGSMSSRIESLTIQEDAKEGVEDEEDLPATGGGGGHPYDRLKTTSTDPVSDIDV
TRREAYLSSEEFKEKFGMTKEAFYKLPKWKQNKFKMAVQLF

14. **G$_5$-HP60** *(generated via sortase-mediated cleavage of IDR-G$_5$-HP60)*
GGGGGHPYDRLKTTSTDPVSDIDVTRREAYLSSEEFKEKFGMTKEAFYKLPKWKQNKFKMAVQLF
