# Supplementary material for: Sortase-mediated segmental labeling: A method for segmental assignment of intrinsically disordered regions in proteins
Source: PLoS One. 2021 Oct 28;16(10):e0258531. doi: 10.1371/journal.pone.0258531 (PMC8553144; doi:10.1371/journal.pone.0258531)

S4 Figure

A

001 MSVSMRDLDP AFQGAGQKAG IEIWRIENFI PTPIPKSSIG KFFTGDSYIV
051 LKTTALKTGA LRHDIHYWLG KDTSQDEAGT AAVKTVELDA ALGGRAVQYR
101 EVQGHETEKF LSYFKPCIIP QEGGVASGFK HVVAEEHITR LFVCRGKHVV
151 HVKEVPFARS SLNHDDIYIL DTKSKIFQFN GSNSSIQERA KALEVVQYIK
201 DTYHDGTCEV ATVEDGKLMA DADSGEFWGF FGGFAPLPRK TANDEDKTYN
251 SDITRLFCVE KGQANPVEGD TLKREMLDTN KCYILDCGIE VFVWMGRTTS
301 LDDRKIASKA AEEMIRSSER PKSQMIRIIE GFETVPFRSK FESWTQETNT
351 TVSEDGRGRV AALLQRQGVN VRGLMKAAPP KEEPQVFIDC TGNLQVWRVN
401 GQAKTLLQAA DHSKFYSGDC YVFQYSYPGE EKEEVLIGTW FGKQSVEEER
451 GSAVSMASKM VESMKFVPAQ ARIYEGKEPI QFFVIMQSFI VFKGGISSGY
501 KKYIAEKEVD DDTYNENGVA LFRIQGSGPE NMQAIQVDPV AASLNSSYYY
551 ILHNDSSVFT WAGNLSTATD QELAERQLDL IKPNQQSRAQ KEGSESEQFW
601 ELLGGKAEYS SQKLTKEPER DPHLFSCTFT KEVLKVTEIY NFTQDDLMTE
651 DIFIIDCHSE IFVWVGQEVV PKNKLLALTI GEKFIEKDSL LEKLSPEAPI
701 YVIMEGGEPS FFTRFFTSWD SSKSAMHGNS FQRKLKIVKN GGTPVADKPK
751 RRTPASYGGR ASVPDKSQQR SRSMSFSPDR VRVRGRSPAF NALAATFESQ
801 NARNLSTPPP VVRKLYPRSV TPDSSKFAPA PKSSAIASRS ALFEKIPPQE
851 PSIPKPVKAS PKTPE*SPAPE SNSKEQ*EEKK ENDKEEGSMS SRIESLTIQE
901 DAKEGVEDEE DLPAHPYDRL KTTSTDPVSD IDVTRREAYL SSEEFKEKFG
951 MTKEAFYKLP KWKQNKFKMA VQLF

B

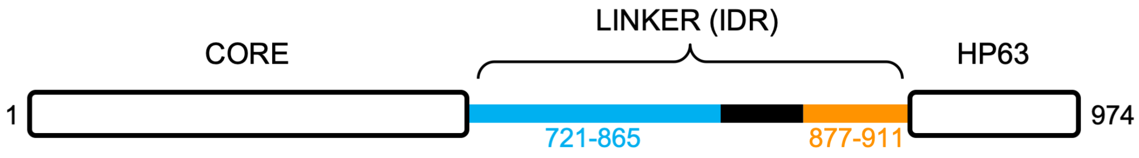

Supplement: S4 Fig — (A) Sequence of A. thaliana villin 4. Positions underlined: V6 domain (621–720) as predicted by homology to V6 domain of chicken villin and HP63 domain (912–974) as determined previously [54, 67]. Positions 721–911 between V6 and HP63 domains represent the disordered linker. (B) Schematic representation of villin 4 domains and linker. The N-terminal core fragment (domains V1 through V6, positions 1–720) and C-terminal headpiece domain (HP63) are represented as boxes. The linker (positions 721–911) is shown as a line. The linker has N-terminal basic (positions 721–865, pI 11.5, shown in blue) and C-terminal acidic (877–911, pI 4.1, shown in orange) regions. A predicted PEST motif (866–876, black italics in panel A) separates the basic and acidic regions of the linker [55, 56]. For the purposes of the experiments in this study, the C-terminal portion of the villin 4 sequence (residues 877–974) was used. (PDF) [file pone.0258531.s004.pdf]
