# Supplementary material for: Sortase-mediated segmental labeling: A method for segmental assignment of intrinsically disordered regions in proteins
Source: PLoS One. 2021 Oct 28;16(10):e0258531. doi: 10.1371/journal.pone.0258531 (PMC8553144; doi:10.1371/journal.pone.0258531)
Supplement: S12 Fig — 15N-HSQC spectra of isolated G5-HP60 (solid red contours, uniform 15N labeling) at (A) 25°C and (B) 5°C. For comparison, each spectrum of G5-HP60 is overlaid with the 15N-HSQC spectrum of atVHP60 (25°C, open blue contours, atVHP60 spectrum reproduced from Miears et al.) [54]. The spectra were recorded at 500 MHz (1H frequency). Circled resonances correspond to amino acid side chains. The sequences of G5-HP60 and atVHP60 are shown at the bottom (coloration matches the corresponding signals in the overlaid spectra). Underlined residues correspond to non-native amino acids added to the HP60 domain. In the case of G5-HP60, five glycines were included to ensure accessibility of the N-terminus for potential sortase-mediated ligation. (PDF) [file pone.0258531.s012.pdf]

S12 Figure

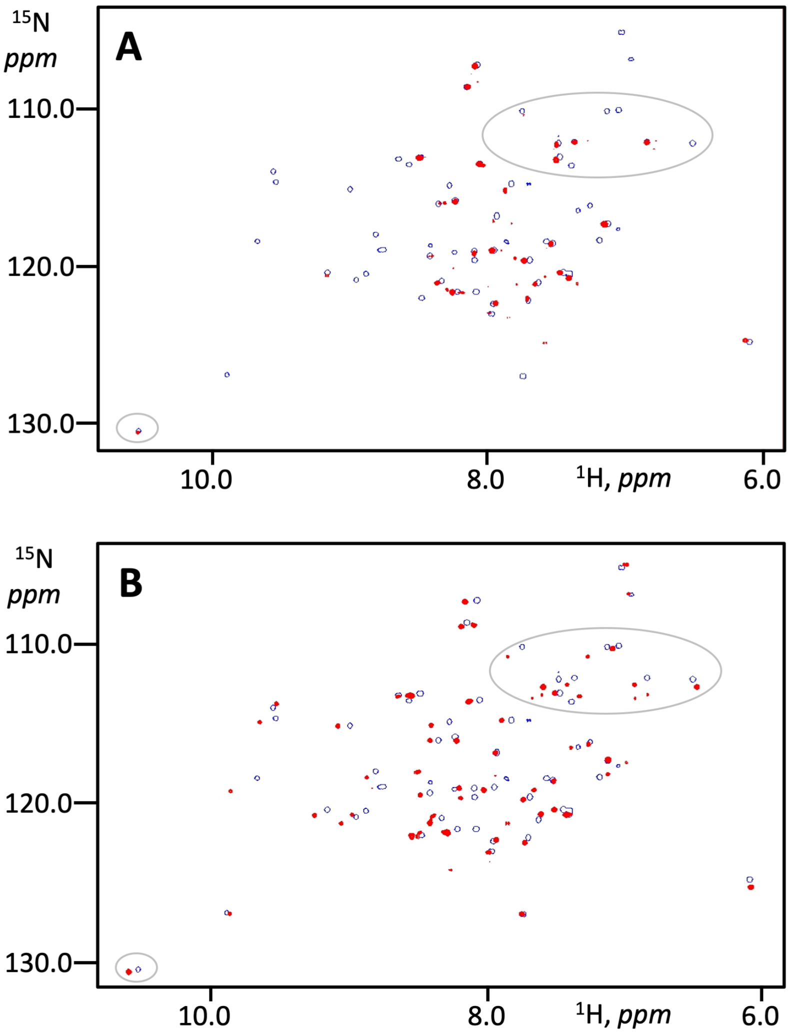

G$_5$-HP60:

GGGGGHPYDR LKTTSTDPVS DIDVTRREAY LSSEEFKEKF GMTKEAFYKL PKWKQNKFKM AVQLF

atVHP60:

HHHHHHPYDR LKTTSTDPVS DIDVTRREAY LSSEEFKEKF GMTKEAFYKL PKWKQNKFKM AVQLF
