# Supplementary material for: Sortase-mediated segmental labeling: A method for segmental assignment of intrinsically disordered regions in proteins
Source: PLoS One. 2021 Oct 28;16(10):e0258531. doi: 10.1371/journal.pone.0258531 (PMC8553144; doi:10.1371/journal.pone.0258531)
Supplement: S1 Table — aCalculated values are average molecular weight predicted using the BMRB Molecular Mass Calculator [68]. For isotopically labeled samples, calculated masses assume 100% incorporation of 15N. bA secondary mass peak of lower intensity was observed at 18158 Da, which is consistent with the presence of a glutathione adduct (calcd mass for glutathione adduct = 18157 Da) [69]. No evidence was found that this adduct impeded SML reactions, and this enzyme preparation was used without additional purification. cMass of FH8-IDR-G doubly labeled with 13C and 15N was not determined. (PDF) [file pone.0258531.s015.pdf]

S1 Table

ESI-MS Characterization of Unlabeled and Uniformly Labeled (^{15}N) Proteins.

Protein	Isotopic Labeling	Calcd (Da)[a]	Obs (Da)
$SrtA_{7M}$	-	17851	17852[b]
FH8-IDR	-	14005	14007
FH8-IDR-G	-	14062	14065
FH8-IDR-G[c]	^{15}N	14232	14217
FH8-IDR-G_3	-	14176	14178
FH8-IDR-$(G_4S)_2$	-	14636	14635
FH8-EDEED	-	11257	11255
IDR-HP(877-974)	^{15}N	12587	12558
GG-HP63	-	7602	7601
GG-HP63	^{15}N	7690	7682
FH8	^{15}N	8675	8669
FH8-IDR-HP63	^{15}N	20947	20927
IDR-HP63	^{15}N	12202	12196

[a]Calculated values are average molecular weight predicted using the BMRB Molecular Mass Calculator. For isotopically labeled samples, calculated masses assume 100% incorporation of ^{15}N.
[b]A secondary mass peak of lower intensity was observed at 18158 Da, which is consistent with the presence of a glutathione adduct (calcd mass for glutathione adduct = 18157 Da). No evidence was found that this adduct impeded SML reactions, and this enzyme preparation was used without additional purification.
[c]Mass of FH8-IDR-G doubly labeled with ^{13}C and ^{15}N was not determined.
