# Supplementary material for: Sortase-mediated segmental labeling: A method for segmental assignment of intrinsically disordered regions in proteins
Source: PLoS One. 2021 Oct 28;16(10):e0258531. doi: 10.1371/journal.pone.0258531 (PMC8553144; doi:10.1371/journal.pone.0258531)
Supplement: S2 Table — aResidue number based on position in the FH8-IDR-HP63 construct. Value in parentheses indicates the corresponding position in wild-type villin 4. The native 35-residue portion of the IDR covers position E79 (E877)–D113 (D911). bEffect of FH8 assessed by comparison of 15N-HSQC spectra for FH8-IDR-HP63 (13C/15N-labeling restricted to FH8-IDR segment, as in Fig 5A) and IDR-HP63 (15N-labeling restricted to IDR segment, as in Fig 5B). All spectra recorded at 25°C. cEffect of GLPETGG insertion assessed by comparison of spectra for FH8-IDR-HP63 and IDR-HP(877–974) (uniformly 15N-labeled, as in S11 Fig). All spectra recorded at 25°C. dObserved chemical shift differences attributed to the presence of the His6-tag on the N-terminus of IDR-HP(877–974) as opposed to the non-native, N-terminal G78 residue in the case of IDR-HP63. eNearly no effect indicates chemical shift differences of <0.04 ppm for the 1H dimension and <0.2 ppm for the 15N dimension. fAlignment of relevant sequences (35-residue IDR fragment indicated in bold). (PDF) [file pone.0258531.s016.pdf]

S2 Table

Summary of ^{15}N-HSQC spectral changes attributed to the presence of the FH8 tag and non-native residues (GLPETGG) inserted for sortase-mediated ligation.

IDR Residue[a]	Effect of FH8[b] (FH8-IDR-HP63 vs IDR-HP63)[f]	Effect of GLPETGG[c] (IDR-HP(877-974) vs IDR-HP63)[f]
E79 (E877) – E80 (E878)	Major change of position and/or intensity[d]	Nearly no effect[e]
K81 (K879)	^{1}H: <0.1 ppm	^{1}H: <0.5 ppm[b]
K82 (K880)	^{1}H: <0.1 ppm; ^{15}N: ~0.2 ppm	^{1}H: <0.5 ppm[b]
E83 (E881)	^{1}H: <0.05 ppm; ^{15}N: <0.2 ppm	Nearly no effect
N84 (N882) – G89 (G887)	Nearly no effect	Nearly no effect
S90 (S888), M91 (M889), S92 (S890), S93 (S891)	unassigned residues	
R94 (R892) (weak signal)	^{1}H: <0.05 ppm; ^{15}N: ~0.3 ppm	Nearly no effect
I95 (I893) – E102 (E900)	Nearly no effect	Nearly no effect
D103 (D901)	unassigned residue	
A104 (A902) – E109 (E907)	Nearly no effect	Nearly no effect
D110 (D908), E111 (E909), E112 (E910)	unassigned residues	
D113 (D911)	Nearly no effect	Nearly no effect

[a]Residue number based on position in the FH8-IDR-HP63 construct. Values in parentheses indicate the corresponding position in wild-type villin 4. The native 35-residue portion of the IDR covers positions E79 (E877) – D113 (D911).
[b]Effect of FH8 assessed by comparision of ^{15}N-HSQC spectra for FH8-IDR-HP63 (^{13}C/^{15}N-labeling restricted to FH8-IDR segment, as in **Fig 5A**) and IDR-HP63 (^{15}N-labeling restricted to IDR segment, as in **Fig 5B)**. All spectra recorded at 25 °C.
[c]Effect of GLPETGG insertion assessed by comparison of spectra for IDR-HP63 (^{15}N-labeling restricted to IDR segment, as in **Fig 5B**) and IDR-HP(877-974) (uniformly ^{15}N-labeled, as in **Figs S9** and **S11**). All spectra recorded at 25 °C.
[d]Observed chemical shift differences attributed to the presence of the His_6-tag on the N-terminus of IDR-HP(877-974) as opposed to the non-native, N-terminal G78 residue in the case of IDR-HP63.
[e]*Nearly no effect* indicates chemical shift differences of <0.04 ppm for the ^{1}H dimension and <0.2 ppm for the ^{15}N dimension.
[f]Alignment of relevant sequences (35-residue IDR fragment indicated in **bold**):

```
FH8-IDR-HP63      PSVQEVEKLLHVLDRNGDGKVSAEELKAFADDSKCPLDSNKIKAFIKEHDKNKDGKLDLKE
IDR-HP63          -------------------------------------------------------------
IDR-HP(877-974)   -------------------------------------------------------------

FH8-IDR-HP63      LVSILSSGTSENLYFQGEEKKENDKEEGSMSSRIESLTIQEDAKEGVEDEEDGLPETGGLP
IDR-HP63          ----------------GEEKKENDKEEGSMSSRIESLTIQEDAKEGVEDEEDGLPETGGLP
IDR-HP(877-974)   ----------MHHHHHHEEKKENDKEEGSMSSRIESLTIQEDAKEGVEDEED-------LP

FH8-IDR-HP63      AHPYDRLKTTSTDPVSDIDVTRREAYLSSEEFKEKFGMTKEAFYKLPKWKQNKFKMAVQLF
IDR-HP63          AHPYDRLKTTSTDPVSDIDVTRREAYLSSEEFKEKFGMTKEAFYKLPKWKQNKFKMAVQLF
IDR-HP(877-974)   AHPYDRLKTTSTDPVSDIDVTRREAYLSSEEFKEKFGMTKEAFYKLPKWKQNKFKMAVQLF
```
